# Single-Cell Genomics of Novel Actinobacteria With the Wood–Ljungdahl Pathway Discovered in a Serpentinizing System

**DOI:** 10.3389/fmicb.2020.01031

**Published:** 2020-06-09

**Authors:** Nancy Merino, Mikihiko Kawai, Eric S. Boyd, Daniel R. Colman, Shawn E. McGlynn, Kenneth H. Nealson, Ken Kurokawa, Yuichi Hongoh

**Affiliations:** ^1^Earth-Life Science Institute, Tokyo Institute of Technology, Tokyo, Japan; ^2^Department of Earth Sciences, University of Southern California, Los Angeles, CA, United States; ^3^Biosciences and Biotechnology Division, Lawrence Livermore National Laboratory, Livermore, CA, United States; ^4^School of Life Sciences and Technology, Tokyo Institute of Technology, Tokyo, Japan; ^5^Graduate School of Human and Environmental Studies, Kyoto University, Kyoto, Japan; ^6^Department of Microbiology and Immunology, Montana State University, Bozeman, MT, United States; ^7^Biofunctional Catalyst Research Team, RIKEN Center for Sustainable Resource Science, Saitama, Japan; ^8^Blue Marble Space Institute of Science, Seattle, WA, United States; ^9^Department of Informatics, National Institute of Genetics, Shizuoka, Japan

**Keywords:** serpentinization, single-cell genomics, *Actinobacteria*, subsurface, alkaliphile, hydrogenase

## Abstract

Serpentinite-hosted systems represent modern-day analogs of early Earth environments. In these systems, water-rock interactions generate highly alkaline and reducing fluids that can contain hydrogen, methane, and low-molecular-weight hydrocarbons-potent reductants capable of fueling microbial metabolism. In this study, we investigated the microbiota of Hakuba Happo hot springs (∼50°C; pH∼10.5–11), located in Nagano (Japan), which are impacted by the serpentinization process. Analysis of the 16S rRNA gene amplicon sequences revealed that the bacterial community comprises *Nitrospirae* (47%), “Parcubacteria” (19%), *Deinococcus-Thermus* (16%), and *Actinobacteria* (9%), among others. Notably, only 57 amplicon sequence variants (ASV) were detected, and fifteen of these accounted for 90% of the amplicons. Among the abundant ASVs, an early-branching, uncultivated actinobacterial clade identified as RBG-16-55-12 in the SILVA database was detected. Ten single-cell genomes (average pairwise nucleotide identity: 0.98–1.00; estimated completeness: 33–93%; estimated genome size: ∼2.3 Mb) that affiliated with this clade were obtained. Taxonomic classification using single copy genes indicates that the genomes belong to the actinobacterial class-level clade UBA1414 in the Genome Taxonomy Database. Based on metabolic pathway predictions, these actinobacteria are anaerobes, capable of glycolysis, dissimilatory nitrate reduction and CO_2_ fixation *via* the Wood–Ljungdahl (WL) pathway. Several other genomes within UBA1414 and two related class-level clades also encode the WL pathway, which has not yet been reported for the *Actinobacteria* phylum. For the Hakuba actinobacterium, the energy metabolism related to the WL pathway is likely supported by a combination of the Rnf complex, group 3b and 3d [NiFe]-hydrogenases, [FeFe]-hydrogenases, and V-type (H^+^/Na^+^ pump) ATPase. The genomes also harbor a form IV ribulose 1,5-bisphosphate carboxylase/oxygenase (RubisCO) complex, also known as a RubisCO-like protein, and contain signatures of interactions with viruses, including clustered regularly interspaced short palindromic repeat (CRISPR) regions and several phage integrases. This is the first report and detailed genome analysis of a bacterium within the *Actinobacteria* phylum capable of utilizing the WL pathway. The Hakuba actinobacterium is a member of the clade UBA1414/RBG-16-55-12, formerly within the group “OPB41.” We propose to name this bacterium ‘*Candidatus* Hakubanella thermoalkaliphilus.’

## Introduction

The serpentinization reaction is fundamental to one of the leading hypotheses regarding the emergence of life on Earth, known as the submarine alkaline hydrothermal vent model (Russell et al., 2010; Branscomb and Russell, 2018). It follows that contemporary serpentinite-hosted systems might provide a window into early life. This model is based on the formation of highly reduced products (e.g., H_2_, CH_4_, and formate) from the hydration of ferromagnesian minerals in mafic and ultramafic rocks (e.g., olivine), which are subsequently mixed with solutes in comparatively more oxidized early Earth ocean waters. The resulting geochemical disequilibria could have been an energy source for the formation of early life. Importantly, this combination of alkaline pH and elevated H_2_ concentrations of systems undergoing active serpentinization has been suggested to help overcome key biochemical bottlenecks in autotrophic metabolism, including that of acetogens and methanogens (Boyd et al., 2020), two groups of organisms commonly argued to be among the earliest evolving (Martin and Russell, 2006).

The modern-day analog of this system includes terrestrial serpentinite-hosted ecosystems, or ophiolites, created by the obduction of the oceanic lithosphere thrust onto the continental plate (Nicolas, 2012). Ophiolites are markers for the early oceanic crust, with ages ranging from 2 to 0.6 Ga (Condie, 2016). Moreover, ophiolitic terranes can be several kilometers thick (Condie, 2016), providing access to subsurface life that can persist in these reducing and alkaline (pH > 10) environments. Several studies have examined the microbial communities present in serpentinite-influenced environments, including in the Samail ophiolite (Rempfert et al., 2017; Fones et al., 2019), the Cedars (Suzuki et al., 2013), the Cabeço de Vide Aquifer (Tiago and Veríssimo, 2013), the Coast Range Ophiolite Microbial Observatory (Crespo-Medina et al., 2014; Twing et al., 2017), the Voltri Massif (Quéméneur et al., 2015; Brazelton et al., 2017), and the Zambales ophiolite (Meyer-Dombard et al., 2018). Although these can be distant locations from each other, Meyer-Dombard et al. (2018) identified a ‘principal community’ amongst serpentinizing environments, consisting of key members in the phyla *Firmicutes* (e.g., *Dethiobacter* sp.) and *Proteobacteria* (e.g., *Serpentinomonas* sp.).

The microbial communities of the Hakuba Happo hot spring (36°42′N, 137°48′E) ophiolite located along the Itoigawa–Shizuoka Tectonic Line in central Honshu, Japan have yet to be investigated. This region consists of an ultramafic rock body that is ∼580 Ma old (Sato et al., 2019) and has ongoing serpentinization activity (Suda et al., 2014, 2017). The geochemistry of the site is characteristic of a serpentinite-hosted system, with highly alkaline waters (pH > 10.6) and high concentrations of dissolved H_2_ (201–664 μM) and CH_4_ (124–201 μM) (Suda et al., 2014). The source of H_2_ is likely derived from ‘low’ temperature serpentinization reactions occurring at ∼50°C (Mayhew et al., 2013) while CH_4_ could be from abiotic or biotic origins (Suda et al., 2014). Two wells (well #1 and #3) have been drilled into the Hakuba Happo ophiolite that permit acquisition of subsurface fluids for geochemical and microbiological analyses.

In the present study, we obtained single-cell genomes of an early-branching, uncultivated actinobacterial lineage from Hakuba Happo well #3 (abbreviated hereafter Happo #3), which were among the dominant taxa found in the bacterial community based on 16S rRNA gene amplicon sequences. This actinobacterial lineage was previously designated as RBG-16-55-12 in the SILVA database (Quast et al., 2012; Yilmaz et al., 2014) and approximately corresponds to the UBA1414/RBG-13-55-18/UBA9087 clade in the Genome Taxonomy Database (GTDB) (Parks et al., 2018). Herein, we predict the metabolic properties and provide the first detailed genome analysis of a bacterium in the clade UBA1414/RBG-13-55-18/UBA9087. This comes two decades after the discovery of its presence by 16S rRNA gene sequencing analysis from samples collected at Obsidian Pool in Yellowstone National Park where it acquired the name “OPB41” (Hugenholtz et al., 1998).

## Materials and Methods

### Sample Collection and Geochemical Measurements

Samples were collected from Happo #3 (36°42’48.6″N 137°48’26.3″E) in October 2016. Detailed geochemical analysis of Happo #3 was previously described in Suda et al. (2014), including isotope compositions and ion concentrations. Happo #3 is a drilling well that extends to about 700 m depth and water is pumped to the surface for the hot spring facilities provided in Happo Town, Japan (Suda et al., 2014). In the field, water temperature (water resistant thermometer CT-430WP, CUSTOM, Japan), pH (pH meter model D-51 with electrode 9625-10D and B-712, HORIBA, Japan), oxidation-reduction potential (ORP; ORP meter model RM-30P with electrode PST-2739C, TOA-DKK, Japan), dissolved oxygen (DO; DO meter model DO-31P with electrode OE-270AA, Japan), electrical conductivity (EC; EC meter model CM-31P with electrode CT-27112B, Japan), salinity (B-721 meter, HORIBA, Japan), calcium (B-751 meter, HORIBA, Japan), sodium (B-722 meter, HORIBA, Japan), and potassium (B-731 meter, HORIBA, Japan) ion concentrations were measured. Analysis of ions and organic acids are described in the Supplementary Information.

Happo #3 water was filtered using two different methods (“Total” and “Sequential”) at a flow rate of about 15 mL per min for 22 h (total water filtered ∼ 19.8 L). For the “Total” method, a 0.1 μm Omnipore membrane (25 mm diameter, Millipore, United States) was used, while the “Sequential” method used in-series filtration consisting of a 0.22 μm Sterivex-GP (polyethersulfone, Millipore, United States), followed by a 0.1 μm Omnipore membrane. The Omnipore membrane was housed in a PerFluoroAlkoxy filter holder (Advantec, United States). Filtered samples were aseptically placed in 100 μL of fresh glycerol-Tris-EDTA buffer (Rinke et al., 2014) for single-cell genomics. Glycerol-Tris-EDTA consisted of 20 mL TE buffer (100×, pH 8) and 100 mL glycerol per 180 mL, which was sterilized by passing through a 0.1 μm filter. Samples were immediately shipped at −20°C overnight and stored at −80°C.

### 16S rRNA Gene Amplicon Sequencing

For 16S rRNA gene amplicon sequencing, another set of filters was collected as described above. DNA was extracted from the “Total” and “Sequential” samples, using the ZymoBIOMICS DNA/RNA Miniprep Kit (Zymo Research, United States). The V3–V4 region of the 16S rRNA genes was amplified by PCR with primers 341F (5’-CCTACGGGNGGCWGCAG) and 785R (5’-GACTACHVGGGTATCTAATCC) according to the Illumina MiSeq Protocol “16S Metagenomic Sequencing Library Preparation,” and the amplicons were used for preparation of sequencing libraries with the KOD FX Neo Kit (Toyobo Life Science, Japan). Sequencing was performed using the Illumina MiSeq platform with the V3 reagent kit (600 cycles). A total of 17,058 (“Total”) and 10,390 (“Sequential”) reads were obtained after quality filtering and trimming via DADA2 (Callahan et al., 2016). The reads were sorted to amplicon sequence variants (ASV), or unique sequences, using DADA2 and taxonomically identified (Callahan et al., 2016, 2017). Afterward, phyloseq v1.26.1 (McMurdie and Holmes, 2013) was used to prune the samples of ASVs observed in a negative control of filtered air collected during field sampling. For the remaining ASVs, a prevalence threshold of 0.1 was determined by phyloseq.

### Single-Cell Sorting, Whole Genome Amplification, and Library Preparation

A fluorescence-activated cell sorter (FACS; BD FACS Aria IIU, BD Biosciences, United States) with a 70 μm nozzle orifice was used to sort single cells into 96-well plates. Filters stored in glycerol-Tris-EDTA stock were thawed on ice and briefly shaken to re-suspend cells from the filter, and 0.65 μL of 1 g/L FM^TM^ 1-43FX (Thermo Fisher Scientific, United States) was then added to an aliquot (350 μL) to stain the cell membrane. The sample was incubated for at least 15 min on ice and was not pre-screened through a 70 μm mesh-size cell strainer (BD Biosciences, United States) to prevent the loss of microbial cells since the Happo #3 water did not contain large particles or microorganisms > 70 μm. The FACS sorting operating condition was checked by calibrating against the BD CS&T Beads (BD Biosciences, United States). A total of 5 plates were sorted for “Total” filters and 8 plates for “Sequential” filters. FACS parameters are further described in Supplementary Information. Targeted cells were sorted into 96-well plates with 2 wells reserved for whole genome amplification (WGA) positive controls (with added template DNA) and 8 wells were reserved for the negative control (without droplet deposition). Each plate was immediately placed at −80°C until processed. Several single-cell lysis methods were tested and described in the Supplementary Information. For WGA, the Qiagen REPLI-g Single Cell Kit (Qiagen, Germany) was used with a modified protocol, as described in the Supplementary Information.

The WGA products were diluted (5 μL WGA product, 95 μL UV-sterilized H_2_O), mixed by pipetting 15 times, and 1 μL was used in a qPCR reaction (SsoAdvanced^TM^ Universal SYBR^®^ Green Supermix, Bio-Rad Laboratories, United States) to amplify the 16S rRNA gene V6–V8 hypervariable regions with primers 926wF and 1392R (Rinke et al., 2014). The qPCR reaction contained 5 μL SsoAdvanced^TM^ Supermix, 0.2 μL forward primer (10 μM stock), 0.2 μL reverse primer (10 μM stock), 3.6 μL UV-sterilized H_2_O, and 1 μL of the diluted WGA product. The qPCR reaction cycle comprised 98°C for 3 min, 35 cycles of 98°C for 15 s and 60°C for 1 min, a melt curve of 95°C for 15 s, 60°C for 1 min, with ramp of +0.3°C to 95°C for 15 s, followed by a 4°C hold. Amplification of 16S rRNA genes was confirmed by gel electrophoresis, and 5 μL of qPCR products were treated with 2 μL ExoSAP-IT Express (ThermoFisher Scientific, United States). The cleaned qPCR products were then sent for Sanger sequencing with primer 1392R to enable cell selection for sequence library preparation. Supplementary Table S1 describes the cells selected for sequencing, including FACS conditions, lysis and WGA reaction conditions, and single-cell genome statistics referenced against the minimum information of single amplified genome (MISAG) criteria (Bowers et al., 2017). Libraries were prepared using the TruSeq DNA PCR-Free Library Preparation Kit (Illumina, United States) and a Covaris M220 to obtain 550 bp sheared DNA.

### Sequencing, Assembly, Binning, and Annotation

All single-cell amplified genome (SAG) libraries were sequenced on the Illumina MiSeq platform using 2 × 300 bp paired-end sequencing (MiSeq v3 Reagent Kit). Raw reads were evaluated using FastQC v0.11.5^1^ and trimmed and quality filtered by Trim_galore! v0.4.1^2^, which uses the cutadapt v1.9.1 program (Martin, 2011). Trim_galore! parameters were set for paired-end files and included a stringency of 5, e 0.1 (error rate), q 20,20 (quality), with the option to retain unpaired reads. Reads were then assembled with SPAdes v3.10.1 (Bankevich et al., 2012) for single-cell samples with the “careful” option and default parameters (k-mers: 21, 33, and 55). Scaffold names were simplified for the Anvi’o v5.3 workflow (Eren et al., 2015), followed by read-mapping with Bowtie2 v2.3.2 (Langmead and Salzberg, 2012) (parameters very-sensitive-local and dovetail) with the samtools depth function to determine coverage values by searching the trimmed reads against the assembled scaffolds. The Anvi’o workflow was then used to cluster and profile the scaffolds greater than 1,000 bp and potential contaminants were removed. The ACDC program (Lux et al., 2016) was also used for contamination screening. Subsequently, gene identification was conducted using Prodigal v2.6.2 (Hyatt et al., 2010) and HMMER v3.1b2^3^. Functional classification was conducted using InterProScan v5.28-67.0 (with databases: TIGRFAMs, SFLD, HAMAP, ProSiteProfiles, ProSitePatterns, PANTHER, Pfam, CDD) (Jones et al., 2014) and imported into Anvi’o. Secondary metabolite biosynthetic gene clusters were identified using antiSMASH v4.1.0 with the options –clusterblast –subclusterblast –knownclusterblast –smcogs –inclusive –borderpredict –full-hmmer –asf –tta (Blin et al., 2017). MAPLE was used to obtain KEGG orthologous (KO) group assignments (Takami, 2014; Arai et al., 2018). Gas vesicle genes were annotated by using a manually curated gas vesicle hidden Markov model database, which is described in the Supplementary Information.

Taxonomic classification was conducted with Kaiju v1.5.0 (Menzel et al., 2016) against the NCBI non-redundant (nr) database (nr + euk database) and imported into Anvi’o. Prophage regions were detected on contigs > 2,000 bp in PHASTER (Zhou et al., 2011; Arndt et al., 2016), and clustered regularly interspaced short palindromic repeat (CRISPR) and its associated gene (Cas) regions were annotated using CRISPRCasFinder (Couvin et al., 2018). SAG sequences were manually refined through Anvi’o (anvi-interactive). CheckM v1.0.7 (Parks et al., 2015) was also used to estimate completeness, degree of contamination, and strain heterogeneity. The number of rRNA genes was determined by the Anvi’o v5.3 method (Eren et al., 2015) and Barrnap v0.6^4^.

### Co-assembly of SAGs

Ten SAGs (Supplementary Table S1) were subsequently co-assembled using SPAdes v3.10.1 with k-mers that were normalized to achieve a flat coverage distribution (target normalization depth = 100 for k-mers with at least 5 depth coverage) via BBNorm v37.95^5^ using default parameters. A range of k-mers were tested (21, 33, 55, 77, 99, and 127) and scaffolds produced when using k-mers 21 and 33 achieved the highest N50 of 7,442 bp based on Quast v4.5 (Gurevich et al., 2013). The generated scaffolds were subsequently placed into the Anvi’o v5.3 workflow with Bowtie2 v2.3.2 read-mapping, as described above, and after removal of contigs < 1,000 bp and potential contaminants (based on sequence composition clustering), the N50 was 8,580 bp. Functional and taxonomic classification were also conducted as described above. The number of tRNA and rRNA genes were determined using tRNAscan-SE v2.0 (Lowe and Eddy, 1997) and Anvi’o v5.3 or Barrnap v0.6, respectively. Effective DB (Eichinger et al., 2016) was used to predict the fully functional bacterial secretion systems Type III, IV, and VI.

### Phylogenetic and Comparative Genomic Analyses

The co-assembly was then placed into phylogenetic trees with reference genomes from the NCBI RefSeq and GenBank databases (O’Leary et al., 2016). The trees included *Actinobacteria* genomes from Rifle, CO (United States) (Anantharaman et al., 2016), a CO_2_-driven geyser (Colorado Plateau, Utah, United States) (Probst et al., 2018), the Sanford Underground Research Facility (SURF) (Momper et al., 2017), and Baltic Sea sediments (Bird et al., 2019; cleaned assemblies provided by Dr. Karen Lloyd). These genomes were the most closely related to the Hakuba SAGs, as determined by classification using the Genome Taxonomy Database Toolkit v0.2.2 (GTDB-Tk), which is a database of quality-controlled genomes that aims to standardize microbial taxonomy through genome phylogeny (Parks et al., 2018). Pyani v0.2.8^6^ and the enveomics collection toolbox were used to calculate the pairwise average nucleotide identity (ANI) and the pairwise average amino acid identity (AAI) between the genomes, respectively (Konstantinidis and Tiedje, 2005a, b; Rodriguez-R and Konstantinidis, 2014, 2016). The occurrence of split genes was analyzed as described in Supplementary Information. Two phylogenetic reconstructions were conducted to evaluate the phylogenetic placement of the Hakuba *Actinobacteria* genome:

(1) A maximum likelihood (ML) tree was created using the GToTree v1.1.6 (Lee, 2019) pipeline based on 138 *Actinobacteria*-specific single copy genes^7^. Reference genomes from *Actinobacteria* were used, and the outgroups consisted of several genomes from each family of *Firmicutes* and *Proteobacteria* (Supplementary Table S2). The concatenated multiple sequence alignment of deduced amino acids was then uploaded to the CIPRES Science Gateway (Miller et al., 2010) to create a ML tree using RAxML-HPC2 on XSEDE (Stamatakis et al., 2008; Stamatakis, 2014) with options WAG PROTGAMMA model and autoMRE bootstrapping.

(2) A Bayesian phylogenetic reconstruction was conducted in Beast2 v2.5.2 (Bouckaert and Vaughan, 2019) with a subset of reference genomes used for the ML tree reconstruction (Supplementary Table S3). After generating a multiple sequence alignment using GToTree, a Bayesian tree was constructed using the WAG substitution model that assumed a gamma distribution with 4 categories and a relaxed clock log normal distribution with Markov chain Monte Carlo simulations (Drummond et al., 2002) set to 50,000,000 (logging every 5,000). This substitution model was selected with PartitionFinder v2.1.1 (Lanfear et al., 2016). A burn-in of 70 percent was set to combine two converging trees of Beast2, as viewed using Tracer v1.7.1 (Rambaut et al., 2018), resulting in 13,501 samples and an effective sample size of 1,278 for tree likelihood and 529 for posterior.

The phylogenetic placement of the Hakuba *Actinobacteria* co-assembled genome amongst all the genomes available in the NCBI RefSeq and Genbank database was confirmed using GTDB-Tk v0.2.2 (reference database version r86 v3). Taxonomic classification was confirmed with the classify workflow (classify_wf), which utilizes the third-party dependencies pplacer (Matsen et al., 2010), FastANI (Jain et al., 2018), Prodigal (Hyatt et al., 2010), FastTree (Price et al., 2010), and HMMR (Eddy, 2011). The classify workflow will first identify bacterial and archaeal marker genes, followed by creating and concatenating multiple sequence alignments. After filtering the alignment to 5,000 amino acids, the workflow will then classify each genome using the GTDB-Tk reference tree and determine the relative evolutionary divergence and ANI.

Selected Protein Sequences [Carbon Monoxide Dehydrogenase / Acetyl-CoA Synthase (CODH/ACS), formylmethanofuran dehydrogenase (Fwd), V-type ATPase, adenosine-5′-phosphosulfate (APS), 3′-phosphoadenosine 5′-phosphosulfate (PAPS) reductase, nitrate reductase alpha subunit NarG, and RubisCO] were aligned using MAFFT (Katoh et al., 2002) with options –maxiterate 1000 and default parameters. For the proteins CODH/ACS, APS, PAPS, NarG, and RubisCO, phylogenetic trees were created. Briefly, gaps were removed with trimAI v1.4.rev15 (Capella-Gutierrez et al., 2009) using option automated1. Manual curation was also done before creating a ML tree using either FastTree v2 (Price et al., 2010) or RAxML on the CIPRES Science Gateway (Miller et al., 2010). All phylogenetic trees were checked using Archaeopteryx (Han and Zmasek, 2009) and FigTree^8^.

Genes coding for [NiFe]- and [FeFe]-hydrogenases were identified by comparison against a curated in-house database (E.S. Boyd, unpublished data). Resulting catalytic subunits were checked for characteristic N- and C-terminal cysteine motifs associated with [NiFe]-hydrogenase variants and the L1, L2, and L3 motifs for [FeFe]-hydrogenase variants (Vignais and Billoud, 2007). The large catalytic subunits of the [NiFe]-hydrogenases were subjected to phylogenetic analysis, as described above, but using the IQtree ML algorithm with the LG+G amino acid substitution model and 1,000 bootstraps to evaluate node support. The phylogenetic analysis included representatives of the primary [NiFe]-hydrogenase groups (Greening et al., 2016) in addition to close representatives of the query sequences that were present in the NCBI database. Gene neighborhood analysis was conducted by surveying the co-assembly and individual SAGs for representatives associated with either Group 3 [NiFe]-hydrogenases (Peters et al., 2015) or those associated with [FeFe]-hydrogenases (Poudel et al., 2016).

## Results and Discussion

### Bacterial Community Structure of the Hakuba Happo #3 Well

The geochemistry of Happo #3 waters from 2011 to 2016 is summarized in Table 1. The taxonomic composition of the bacterial community based on 16S rRNA gene amplicon sequencing is depicted in Supplementary Figure S1 and summarized in Supplementary Table S4. The dominant bacterial phyla were *Nitrospirae* (47%), “Parcubacteria” (19%), *Deinococcus-Thermus* (16%), and *Actinobacteria* (9%), followed by *Firmicutes* (5%), *Bacteroidetes* (2%), among others (<1%). Only 57 ASVs were detected from both the “Total” (17,058 total reads) and “Sequential” (10,390 total reads) samples, and the majority (90%) were represented by 15 ASVs. Such low bacterial diversity is consistent among serpentinite-hosted systems. For example, at The Cedars, 16 phylotypes (>99% sequence similarity cutoff) represented 84% of the 16S rRNA amplicon sequences recovered from the shallow-sourced spring and 98% of those from the deep-sourced spring (Suzuki et al., 2013). In the Cabeço de Vide Aquifer, 45 phylotypes (≥97% similarity cutoff) were identified, dominated by four major taxonomic classes (Tiago and Veríssimo, 2013). Other serpentinite-hosted systems with comparatively few phylotypes include the Samail ophiolite (Rempfert et al., 2017; Fones et al., 2019), the Coast Range Ophiolite Microbial Observatory (Crespo-Medina et al., 2014; Twing et al., 2017), the Voltri Massif (Quéméneur et al., 2015; Brazelton et al., 2017), and the Zambales ophiolite (Meyer-Dombard et al., 2018). The microbial diversity and abundance of cells were previously shown to be pH-dependent within the Samail ophiolite (Rempfert et al., 2017; Fones et al., 2019). Compared to the ‘principal community’ amongst several serpentinite-hosted systems identified by Meyer-Dombard et al. (2018), Happo #3 contained few *Proteobacteria*, whereas *Nitrospirae* and “Parcubacteria” predominated.

**TABLE 1:** Geochemistry of Happo #3 from 2011 to 2016.

	2011^a^	2016
Temperature	48°C	47.5°C^b^
pH	10.7	10.95^b^
ORP	n.m.	−435 mV^b^
EC	48.3 mS/m	47.7 mS/m^b^
DO	0.59 mg/L	0.10 mg/L^b^
Salinity	0.02%	n.d.^b^ (detection limit 0.1%)
H_2_	201 μM	n.m.
CH_4_	124 μM	n.m.
C_2_H_6_	0.2 μM	n.m.
N_2_	1298 μM	n.m.
Ca^2+^	130 μM	220 μM
K^+^	70 μM	125 μM
Na^+^	1160 μM	1565 μM
NH_3_	n.m.	7 μM
Al_3_^+^	10 μM	n.m.
Li^+^	10 μM	n.m.
Cl^–^	180 μM	155 μM
SO_4_^2–^	10 μM	8 μM
F^–^	4 μM	n.m.
Formate	n.m.	80 μM
Acetate	n.m.	<40 μM
Not detected^c^		Pyruvate, lactate propionate, NO_2_^–^, NO_3_^–^, HCO_3_^–^, Mg^2+^, PO_4_^2–^, total Fe, nucleobases, nucleosides, and amino acids

*^*a*^Published in Suda et al. (2014). ^*b*^Nobu et al., unpublished data. ^*c*^No peak detected; Detection limits: Pyruvate (40 μM), lactate (40 μM), propionate (40 μM), NO_2_^–^ (2.2 μM), NO_3_^–^ (1.6 μM), HCO_3_^–^ (0.02 μM), Mg^2+^ (20.6 μM), PO_4_^2–^ (0.01 μM). CO_2_ and total Fe not detected as described in Suda et al. (2014). Nucleobases and nucleosides include cytosine (179 nM), uracil (659 nM), adenine (740 nM), guanine (1.3 μM), C-ribonucleosides (47 nM), U-ribonucleosides (340 nM), A deoxyribonucleosides (71 nM), G deoxyribonucleosides 64 nM), C-deoxyribonucleosides (470 nM), T-deoxyribonucleosides (133 nM), A-deoxyribonucleosides (400 nM), and CH1 amino acids (50 μM). n.m. = not measured. n.d. = not detected.*

The Happo #3 community included three ASVs affiliated with an early-branching, uncultivated *Actinobacteria* lineage that has not been previously observed in terrestrial serpentinite-hosted systems. These *Actinobacteria* ASVs shared 98% sequence identity and clustered with the clade RBG-16-55-12 in the SILVA v132 database (Quast et al., 2012; Yilmaz et al., 2014), previously classified within the clade OPB41 in the SILVA v128 database. The RBG-16-55-12 members are located in a variety of environments, including subsurface environments (Anantharaman et al., 2016), mine tailing ponds (Ramos-Padrón et al., 2011), mud volcanoes (Chang et al., 2012), hot springs (Hugenholtz et al., 1998), and deep sea sediments (Kato et al., 2009).

### General Characteristics and Taxonomic Classification of the Hakuba Actinobacteria SAGs

We conducted single-cell genomics of the Happo #3 samples and identified 10 SAGs belonging to the RBG-16-55-12 clade based on their 16S rRNA sequences. The general characteristics of these 10 SAGs are listed in Supplementary Table S1. “Low” (*n* = 6), “Medium” (*n* = 3), and “High” (*n* = 1) quality SAGs were identified according to the MISAG standard for *Bacteria* and *Archaea* (Bowers et al., 2017). The range of completeness was between 33.1 and 92.8% with 0.7% and 6.5% contamination (median = 1.4% contamination), as estimated by the Anvi’o marker gene-based approach (Eren et al., 2015; Supplementary Table S5). Based on ANI (Supplementary Table S6) and AAI (Supplementary Table S7), these 10 SAGs represent the same species (≥98% pairwise ANI for all 10 SAGs; ≥ 90% pairwise AAI for SAGs with > 50% completeness) with GC content ranging from 48.5 to 49.2%. It has been suggested that species boundary is approximately 95% (ANI) and 90% (AAI) (Konstantinidis and Tiedje, 2005a; Richter and Rosselló-Móra, 2009).

The genomes were subsequently co-assembled into one composite genome assembly (“Hakuba co-assembly”) of all 10 SAGs combined, resulting in 93.5% completeness and 6.5% contamination (Table 2). The co-assembly was generated to guide genome analysis of the SAGs to supplement the inherent biases of single-cell genomics caused during WGA (e.g., chimeric DNA, uneven genome coverage, low completeness) (Xu and Zhao, 2018). Sequence similarity analysis of the 588 co-assembled contigs using the Kaiju taxonomic classifier (Menzel et al., 2016) with the NCBI nr database did not provide confident placement of the taxonomic position of this genome. The taxonomic affiliation of the contigs was not consistent (Supplementary Table S8): the contigs were affiliated with “unclassified” (43%), *Firmicutes* (11%), *Proteobacteria* (9%), *Actinobacteria* (5%), *Chloroflexi* (4%), *Nitrospirae* (4%), *Euryarchaeota* (2%), “Omnitrophica” (2%), and others (<1%). The contigs not taxonomically identified as *Actinobacteria* were not removed for two reasons: (1) the SAG and co-assembled genome redundancy (Anvi’o) and contamination (CheckM) were < 6.5% (median = 1.4% contamination) (Supplementary Table S1) and (2) contig clustering by sequence composition on Anvi’o and ACDC did not reveal that these taxa contributed to contamination (Supplementary Figure S2). Similar inconsistent results of taxonomic affiliation were reported for genomes of *Bacteria* belonging to deeply branching lineages with limited reference sequences, such as members within the candidate bacterial phylum OP9 (Dodsworth et al., 2013).

**TABLE 2:** Basic information for the co-assembled Hakuba genome and SAG S34.

Analysis project type	Co-assembled genome	SAG ID: S34
DDBJ BioProject	PRJDB8357	PRJDB8357
DDBJ Accession Number Co-assembly	BLSE01000001–BLSE01000587	
DDBJ Accession Number SAGs	BLRU01000000–BLRZ01000000, BLSA01000000–BLSD01000000	BLRZ01000001–BLRZ01000510
**Co-assembly/SAG information**		
Cell isolation approach	FACS	
Single cell lysis approach	Chemical and enzymatic	
Single cell kit	Qiagen REPLI-g Single Cell Kit (multiple displacement amplification)	
Assembly software	SPAdes v3.10.1	
Estimation of completeness	Anvi’o v5.3 (marker gene-based approach)	
Assembly quality^*a*^	Medium quality	High quality
Estimated completeness	93.5%	92.8%
Contamination	6.5%	1.4%
**Genome information**		
Genome size	2,947,136 bp	2,120,563 bp
Number of contigs	588	316
N50	8,580	13,695
Max contig length	37,255 bp	55,032 bp
rRNAs	2 (16S), 2 (23S), 1 (5S)	1 (16S), 1 (23S)
tRNAs	45	47
GC Content	48.5%	48.6%

*The genome was co-assembled from 10 single-cell amplified genomes (SAGs) collected from Happo #3 (see Supplementary Table S1 for individual SAGs). The SAG S34 represents the highest quality SAG obtained. ^*a*^Based on the minimum information of single amplified genome (MISAG) criteria (Bowers et al., 2017).*

Based on taxonomic analysis using GTDB-Tk (Supplementary Table S9), the Hakuba co-assembly and the 10 SAGs were classified to the uncultured, class-level clade “UBA1414” in the *Actinobacteria* phylum. This clade includes four Baltic Sea SAGs and one metagenome-assembled genome (MAG) from the Rifle aquifer (GCA_001767735). GTDB-Tk was also used to estimate the novelty of the Hakuba *Actinobacteria* genomes by calculating a relative evolutionary divergence metric and comparing against the GTDB rank normalized taxonomy. This metric is more robust than pairwise AAI to assign taxonomic rank as it considers the variation in the evolutionary tempos amongst different lineages (Hugenholtz et al., 2016; Parks et al., 2018). Based on this metric, the Hakuba SAGs and co-assembly could represent a new order within the UBA1414 class while the Baltic Sea SAGs and Rifle MAG represent new species within the genus currently named “20-14-0-20-35-9” in GTDB. According to the classification of GTDB, two closely related class-level clades to UBA1414 are “UBA9087” and “RBG-13-55-18,” which consist of several MAGs from the Rifle aquifer, one MAG from Crystal Geyser, and one MAG from SURF (Table 3). These three clades (UBA1414, UBA9087, and RBG-13-55-18) correspond to two 16S rRNA-based clades in the SILVA v132 database: WCHB1-81 and the above-mentioned clade, RBG-16-55-12.

**TABLE 3:** Information for the metagenome-assembled genomes (MAGs) and single-cell amplified genomes (SAGs) analyzed in this paper.

Clade	ID	NCBI BioProject (BioSample)	Completeness (%)	Contamination (%)	References
UBA1414	Baltic Sea_59E_21H_M23	PRJNA417388^a^	54.7	1.4	Bird et al., 2019
	Baltic Sea_59E_21H_O21	PRJNA417388^a^	52.5	1.4	Bird et al., 2019
	Baltic Sea_60B_13H_A10	PRJNA417388^a^	69.8	2.9	Bird et al., 2019
	Baltic Sea_60B_13H_C09	PRJNA417388^a^	71.9	1.4	Bird et al., 2019
	GCA_001767735_Rifle_CO	PRJNA288027	63.3	2.9	Anantharaman et al., 2016
		(SAMN04314056)			
	Hakuba_co-assembly^b^	PRJDB8357	93.5	6.5	This study
RBG-13-55-18	GCA_001767755_Rifle_CO	PRJNA288027	60.4	0.0	Anantharaman et al., 2016
		(SAMN04313673)			
	GCA_001768645_Rifle_CO	PRJNA288027	97.1	0.7	Anantharaman et al., 2016
		(SAMN04313996)			
	GCA_001767605_Rifle_CO	PRJNA288027	97.8	0.0	Anantharaman et al., 2016
		(SAMN04313722)			
	SURF_21	PRJNA355136^c^	89.2	0.7	Momper et al., 2017
UBA9087	GCA_001767575_Rifle_CO	PRJNA288027	78.4	1.4	Anantharaman et al., 2016
		(SAMN04314195)			
	GCA_001871795_Crystal_Geyser	PRJNA297582	95.7	0.7	Burstein et al., 2016
		(SAMN04328288)			

*Completeness and contamination were determined by Anvi’o marker gene-based approach (Eren et al., 2015). ^*a*^ The cleaned genome assemblies were provided by Dr. Karen Lloyd. ^*b*^The highest quality Hakuba SAG S34 has 92.8% completeness and 1.4% contamination. ^*c*^ The cleaned genome assemblies were obtained from the data depository referenced in Momper et al. (2017).*

**FIGURE 1:**
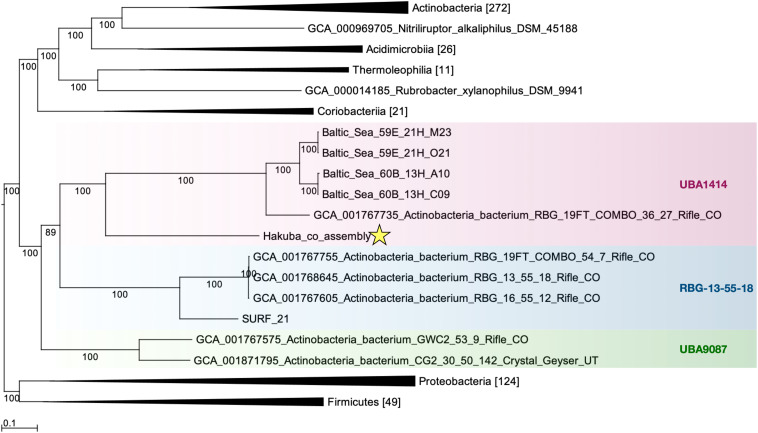
Maximum-likelihood phylogenetic tree of clades UBA1414, RBG-13-55-18, and UBA9087. The Hakuba co-assembly (denoted by a star) formed a clade with the Baltic Sea SAGs and a Rifle MAG. Bootstrap support values are shown on each branch, and the Bayesian posterior probability is depicted in Supplementary Figure S3. Representatives of *Proteobacteria* and *Firmicutes* were used as the outgroups. The genomes used in this figure are listed in Supplementary Table S2 and the number in brackets represents the count of genomes used in the clade. GToTree v1.1.6 (Lee, 2019) and RAxML-HPC2 (Stamatakis et al., 2008; Stamatakis, 2014) on the CIPRES Science Gateway (Miller et al., 2010) were used to create the ML tree, which is based on 138 concatenated *Actinobacteria*-specific single copy genes.

The relationship of the clades UBA1414, UBA9087, and RBG-13-55-18 was further examined by ML and Bayesian phylogenetic analyses of a concatenated protein sequence (Figure 1 and Supplementary Figure S3). The monophyly of the three clades was confirmed by both methods, which generated identical topologies with a high confidence level for the clades. The Hakuba co-assembly formed a clade with the Baltic Sea SAGs and one Rifle MAG (GCA_001767735), similar to the analysis using GTDB-Tk (Supplementary Table S9). However, no genomes showed > 45% pairwise AAI and > 76% pairwise ANI to the Hakuba co-assembly and 10 SAGs (Supplementary Tables S6, S7). It has been suggested that the genus-level boundary is ≥ 60% for pairwise AAI (Rodriguez-R and Konstantinidis, 2014). This further demonstrates the novelty of the Hakuba SAGs, possibly as a new order within the UBA1414 class, as suggested by the GTDB-Tk analysis.

### The Hakuba Actinobacteria Genomes Encode the Wood–Ljungdahl Pathway

**FIGURE 2:**
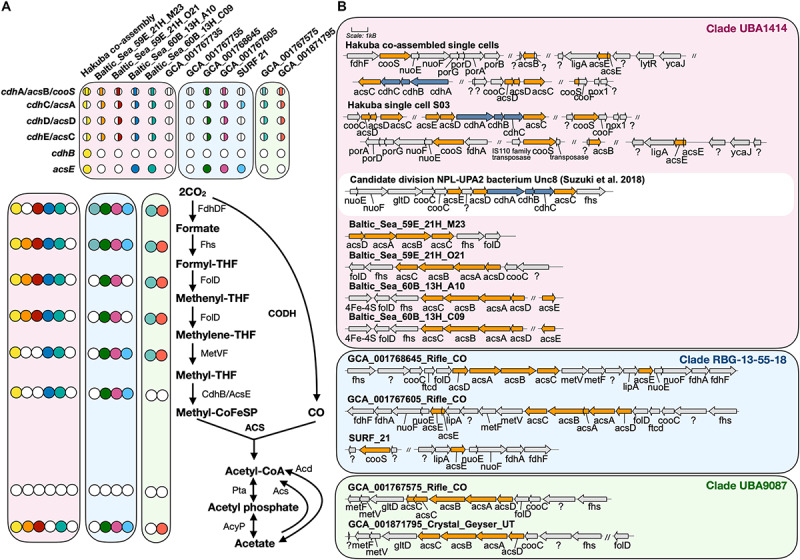
Key enzymes encoded by genomes within clades UBA1414, RBG-13-55-18, and UBA9087 for the Wood–Ljungdahl Pathway. **(A)** The presence of proteins involved in the WL pathway are depicted with a colored circle while the absence is depicted by a white circle. Each color corresponds to a genome used in this study. For CODH/ACS, the colored circle is split by the bacterial- or archaeal-type subunit, and the location of the color indicates whether a gene was present or not within the assembly. The proteins Acs and Acd do not have corresponding circles. **(B)** The gene neighborhood of CODH/ACS is shown for each genome and was designed using Gene Graphics (Harrison et al., 2017). Gene neighborhoods on different contigs are denoted by “//.” The three clades are color coded as done in Figure 1. The gene neighborhood of Candidate division NPL-UPA2 bacterium Unc8 (Suzuki et al., 2018), a putative acetogen from a serpentinite-hosted system called The Cedars, is depicted in a white box for comparison of hybrid CODH/ACS. Abbreviations: *acyP* (acylphosphatase), *acd* (ADP-forming acetyl-CoA synthetase), *acs* (acetyl-CoA synthase), *cdh* (carbon monoxide dehydrogenase), *cooS* (carbon monoxide dehydrogenase), *fdh* (formate dehydrogenase), *fhs* [formyl-tetrahydrofolate (THF) synthase], *folD* (bifunctional 5,10-methenyl-THF cyclohydrolase/5,10-methylene-THF dehydrogenase), *ftcD* (glutamate formiminotransferase/formiminotetrahydrofolate cyclodeaminase), *gltD* (glutamate synthase), *ligA* (DNA ligase), *lipA* (lipoyl synthase), *lytR* (cell envelope-related function), *met* (Methylene-THF reductase), *nox1* (NADH oxidase), *nuo* (NADH-quinone oxidoreductase), *pta* (phosphotransacetylase), *por* (pyruvate ferredoxin oxidoreductase), *ycaJ* (putative ATPase).

The Hakuba co-assembly contains all key genes for the Wood–Ljungdahl (WL) pathway for CO_2_ fixation (Ragsdale, 2008; Figure 2). The presence of this pathway has yet to be reported in *Actinobacteria* (Adam et al., 2018); only genes homologous to CODH (*cooS/cdhA/acsB*) in five actinobacterial genomes have been reported (Inoue et al., 2019). The genes for the WL pathway found in the Hakuba co-assembly are: CODH/acetyl-CoA synthase complex (*acs*), formate dehydrogenase (*fdhDF*), formyl-tetrahydrofolate (THF) synthase (*fhs*), bifunctional 5,10-methenyl-THF cyclohydrolase / 5,10-methylene-THF dehydrogenase (*folD*), and methylene-THF reductase (*metF*). These genes, including those for the CODH/ACS complex, are also found in four other genomes within the clades UBA1414 and RBG-13-55-18, including two genomes from the Baltic Sea (60B_13H_A10 and 60B_13H_C09) and two genomes from Rifle (GCA_001768645 and GCA_001767605).

The Hakuba co-assembly harbors the genes *fdhD* and *fdhF* that encode proteins involved in the reduction of CO_2_ to formate. The source of inorganic carbon could be from the environment or from pyruvate oxidation by the action of pyruvate ferredoxin oxidoreductase (PorABDG) (Ragsdale, 2003) or pyruvate dehydrogenase (PdhABCD) (de Kok et al., 1998). In serpentinite-hosted environments, there is limited dissolved inorganic carbon, and due to the alkaline pH and presence of high concentrations of divalent cations (e.g., Ca^2+^), inorganic carbon, such as CO_2_, is rapidly sequestered into mineral carbonates (Matter and Kelemen, 2009). Indeed, the total inorganic carbon in Happo #3 was undetectable (Suda et al., 2014). One candidate source of inorganic carbon is carbon monoxide (CO). CO can be synthesized in these types of environments (Seewald et al., 2006; McCollom and Seewald, 2007) and has been detected in other serpentinite-hosted systems, such as the Coast Range Ophiolite Microbial Observatory (Twing et al., 2017). Furthermore, CO can be utilized by the microbial community in these ecosystems (Morrill et al., 2014; Fones et al., 2019). The Hakuba co-assembly contains genes for anaerobic-type CO dehydrogenase (*cooS* and *cooF*), and the CO dehydrogenase maturation protein (*cooC*). Bicarbonate, if present, could also be another source of inorganic carbon as the Hakuba co-assembly encodes two Na^+^-dependent bicarbonate transporters, indicating the potential to uptake HCO_3_^–^, similar to acetogens (Braus-Stromeyer et al., 1997; Smith and Ferry, 2000; Pander et al., 2019). However, homologs of genes coding for carbonic anhydrase that converts HCO_3_^–^ to CO_2_ were not detected in the genome assembly.

Besides environmental sources of inorganic carbon, CO_2_ can be generated *via* the action of PorABDG or PdhABCD, as described above. Pyruvate, as the substrate, is produced in glycolysis in this bacterium. Although there are no reports of the abiotic serpentinization reaction resulting in formation of sugars, such as galactose, the Happo #3 well is located in an alpine, forested ecosystem, and the sugars could be derived from soil organic carbon. The utilization of CO_2_ by the WL pathway from pyruvate oxidation *via* glycolysis has been observed in acetogens, such as *Moorella thermoacetica* (Fontaine et al., 1942; Barker and Kamen, 1945; Drake et al., 1981; Menon and Ragsdale, 1996). In such cases, the WL pathway may also work to balance redox and regenerate the electron carriers used during glycolysis or other carbon substrate oxidation (Schuchmann and Müller, 2016).

Genes for the formylmethanofuran dehydrogenase-like complex (*fwdABCD*) are also present within the Hakuba co-assembly or SAGs but without the genes for subunit *fwdE* and the ferredoxin subunits *fwdFG*. The Fwd complex catalyzes the first step in CO_2_ reduction for methanogenesis and contains a tungsten active site (within FwdB), as compared to the molybdenum-dependent isoenzyme Fmd (Hochheimer et al., 1995, 1996; Wagner et al., 2016). The full operon is present in SAG S47 (*fwdDBA-ftr-fwdC*) with formylmethanofuran-tetrahydromethanopterin N-formyltransferase (*ftr*). This operon structure is similar to the organization of a homologous complex, formyltransferase/hydrolase complex (Fhc), found in methylotrophs that converts formyl-H_4_MPT (tetrahydromethanopterin) to formate (Pomper et al., 2002; Adam et al., 2019; Hemmann et al., 2019). However, the genes to synthesize methanopterin derivatives, such as dihydromethanopterin reductase and tetrahydromethanopterin:alpha-L-glutamate ligase (Xu et al., 1999; Maden, 2000; Scott and Rasche, 2002), were not observed in the Hakuba actinobacterium genome. In addition, compared to *fhcB* from the methylotroph *Methylorubrum extorquens* (Hemmann et al., 2019), the amino acid sequence of the catalytic subunit *fwdB* from the Hakuba SAGs lacks the sequence motifs for a tungstopterin cofactor and contains two necessary components for a functioning *fwdB*: a N-terminal domain with [4Fe-4S] cluster and a catalytic Cys118 (Supplementary Figure S4).

In methanogens, FwdABD comprise the catalytic subcomplex while the function of FwdC remains unknown (Wagner et al., 2016). FwdE is an iron-sulfur protein (Hochheimer et al., 1995) and is hypothesized to function as a DNA-binding protein in acetogens (Shin et al., 2016). The gene cluster *fwdABCD* without *fwdE* was also identified in the methanogen OP bin 54 (*Methanomethyliales*) (Berghuis et al., 2019). In *Methanosarcina acetivorans*, the synthesis of FwdDBAC is likely important for carboxydotrophic growth and is potentially involved in the production of formate (Matschiavelli and Rother, 2015). In comparison, in the genomes of 14 cultivated acetogens, only the subunit *fwdE* is observed (Shin et al., 2016). Similarly, the Rifle MAGs within sub-clades RBG-13-55-18 and UBA9087 harbor *fwdE* but not *fwdABCD*. The presence of the catalytic subunits *fwdABD* in the Hakuba co-assembly or SAGs suggests that the Fwd-like complex could be active, with potential function during growth on CO, as demonstrated for *Methanosarcina acetivorans* (Matschiavelli and Rother, 2015).

**FIGURE 3:**
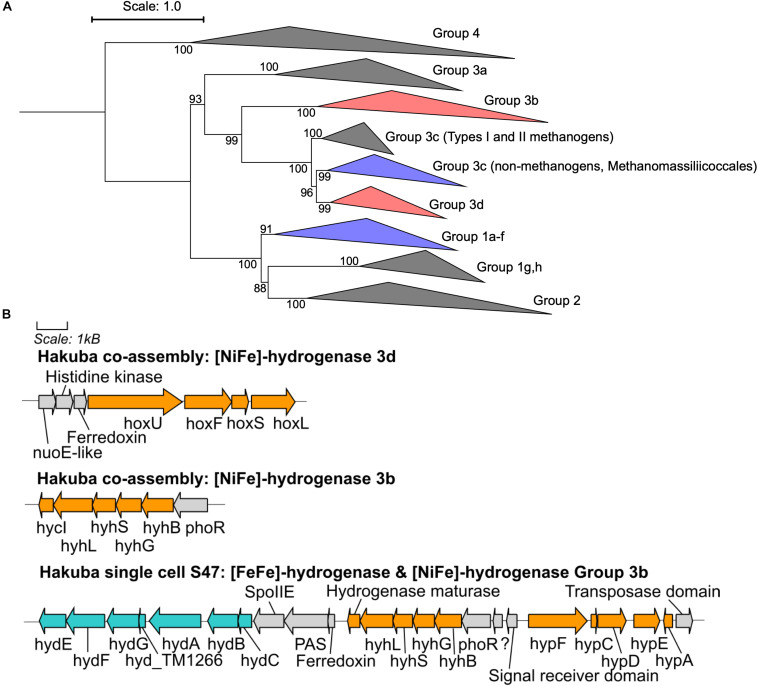
Phylogenetic topology and gene neighborhood of hydrogenases. **(A)** The phylogenetic topology of [NiFe]-hydrogenases for clades UBA1414, RBG-13-55-18, and UBA9087. Groups 3b, 3c, 3d, and 1a-f are further expanded in Supplementary Figures S5, S6. The Hakuba co-assembly is indicated by red color and the other genomes are indicated in blue color. Bootstraps (out of 1000 replicates) are shown at the nodes. **(B)** The gene neighborhood of [NiFe]- and [FeFe]-hydrogenases in the Hakuba co-assembly and SAG S47, as designed using Gene Graphics (Harrison et al., 2017). The genes related to [NiFe]-hydrogenase and hydrogenase maturation are depicted in orange color. The genes related to [FeFe]-hydrogenase and hydrogenase maturation are depicted in blue color. Abbreviations: *hox* (NAD-Coupled [NiFe]-hydrogenase group 3d), *hycI* (hydrogenase maturase), *hydABC* ([FeFe-hydrogenase], *hydEFG* ([FeFe]-hydrogenase maturation), *hyd_TM1266* (putative iron-only hydrogenase system regulator), *hyh* (NADP-coupled [NiFe]-hydrogenase group 3b), *hyp* (hydrogenase expression/formation), *hypA* (hydrogenase nickel incorporation protein), *hypF* (hydrogenase maturation protein F), *nuoE* (NADH dehydrogenase subunit E-like), *phoR* (two-component system; phosphate regulon sensor histidine kinase P), *spoIIE* (Stage II sporulation protein E).

CO_2_ reduction could be driven by reducing equivalents generated by hydrogen (H_2_) oxidation *via* [NiFe]- or [FeFe]-hydrogenases (Figure 3 and Supplementary Figures S5, S6). Although known acetogens encode hydrogenase modules in a gene cluster containing a *fdh* gene (Shin et al., 2016), the hydrogenase genes in genomes of clades UBA1414, UBA9087, and RBG-13-55-18 are not clustered with *fdh*, with the exception of the Rifle MAG GCA_001767575. Considering that these are incomplete genomes derived from metagenomic or single-cell genomic assemblies (Table 3), it is possible that a gene cluster containing *fdh* and hydrogenases was fragmented. Two [NiFe]-hydrogenase genes were identified in the Hakuba co-assembly and were phylogenetically affiliated with Group 3b and Group 3d [NiFe]-hydrogenases (Figure 3A) that are coupled to the bidirectional reduction of NADP^+^ and NAD^+^, respectively, in other organisms (Vignais et al., 2001; Peters et al., 2015). Accordingly, the diaphorase (*hyhG* or *hoxF*) and Fe-S (*hyhB* or *hoxU*) cluster modules associated with these two enzymes were also co-localized with the large (*hyhL* or *hoxL*) and small (*hyhS* or *hoxS*) subunits of the respective [NiFe]-hydrogenase groups (Figure 3B; Peters et al., 2015). The other genome assemblies in the three clades also encoded [NiFe]-hydrogenases that were variably affiliated with either group 1, group 3b, group 3c, or group 3d (Supplementary Figures S5, S6). Amongst all the genome assemblies in the three clades, the Hakuba co-assembly is the only genome to encode a [FeFe]-hydrogenase. The Hakuba co-assembly encoded the catalytic subunit HydA, and the Hakuba SAG S47 also encoded the cluster HydABC along with [FeFe]-hydrogenase accessory proteins, including HydEFG (Posewitz et al., 2004). The HydABC cluster was present within the gene neighborhood of the Group 3b-like [NiFe]-hydrogenase (Figure 3). Homologs of HydABC have been suggested to be involved in electron bifurcation (Schut and Adams, 2009; Poudel et al., 2016), a process where reversible H_2_ oxidation is coupled to simultaneous reduction of NAD^+^ and ferredoxin (Fd) (Buckel and Thauer, 2013). Electron bifurcating hydrogenases are implicated in the energy conservation of model acetogens like *Acetobacterium woodii* (Wiechmann et al., 2020) *via* coupling of Fd (oxidation/reduction) and NAD^+^/NADH reduction and oxidation with H_2_ (oxidation/reduction). It is possible that such activities are also catalyzed by the Hakuba actinobacterial cells in conjunction with the reduction/oxidation of NAD(P)^+^/NAD(P)H via the [NiFe]-hydrogenases.

**FIGURE 4:**
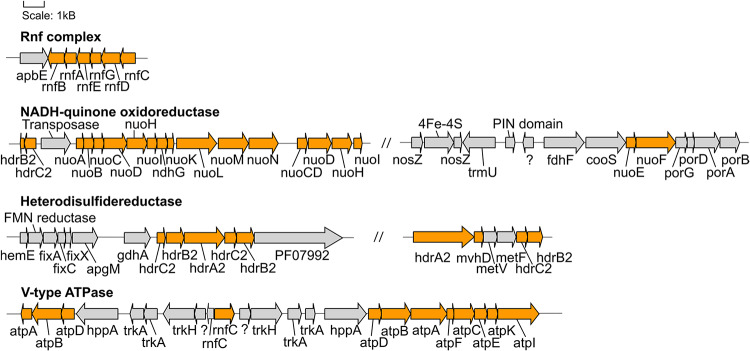
Gene neighborhoods of genes encoding proteins involved in energy conservation found in the Hakuba co-assembly. The genes within the Hakuba co-assembly are colored orange while the other genes are colored gray. Gene neighborhoods on different contigs are denoted by “//.” The gene neighborhood was designed using Gene Graphics (Harrison et al., 2017). Abbreviations: *apbE* (Mg^2+^-dependent flavin transferase), *apgM* (2,3-bisphosphoglycerate-independent phosphoglycerate mutase), *coo* (carbon monoxide dehydrogenase), *fdh* (formate dehydrogenase), *fix* (electron transfer flavoprotein), *gdhA* (glutamate dehydrogenase), *hdr* (heterodisulfide reductase), *hemE* (uroporphyrinogen decarboxylase), *hppA* (K^+^-stimulated pyrophosphate-energized sodium pump), *met* (methylenetetrahydrofolate reductase), *mvhD* (F420-non-reducing hydrogenase iron-sulfur subunit), *ndh* (NAD(P)H-quinone oxidoreductase), *nuo* (NADH-quinone oxidoreductase), *nosZ* (nitrous oxide reductase), *rnf* (Na^+^-translocating ferredoxin:NAD^+^ oxidoreductase), PF07992 (Pyridine nucleotide-disulphide oxidoreductase), *por* (pyruvate ferredoxin oxidoreductase), *trk* (Trk system potassium uptake protein), *trmU* (tRNA-uridine 2-sulfurtransferase).

Over the next few steps of the WL pathway, formate is likely converted to methyl-THF. From formyl-THF to methenyl-THF, the bifunctional 5,10-methenyl-THF cyclohydrolase / 5,10-methylene-THF dehydrogenase (FolD) is likely the enzyme used by the Hakuba bacterium and those within clades UBA1414, UBA9087, and RBG-13-55-18. In comparison, pan-genome analysis of 14 cultivated acetogens demonstrated that this conversion mainly occurs with the enzyme formyl-THF cyclohydrolase (Fch) (Shin et al., 2016). However, in the acetogen *Moorella thermoacetica*, FolD is used as a bifunctional protein with cyclohydrolase and dehydrogenase activity for the two-step conversion of formyl-THF to methylene-THF (O’Brien et al., 1973). Given the absence of *fch* and presence of *folD* in the clades UBA1414, UBA9087, and RBG-13-55-18, these genomes likely contain a bifunctional *folD* similar to *Moorella thermoacetica*. Methylene-THF reductase subunits V and F (MetVF) then catalyze the reduction from methylene-THF to methyl-THF (Figure 4). Interestingly, *metVF* in the Hakuba co-assembly is located with the F_420_-non-reducing hydrogenase iron-sulfur subunit D (*mvhD*) and heterodisulfide reductase subunits *hdrA*, *hdrB*, and *hdrC* (*hdrA-mvhD-metVF-hdrCB*). In comparison, genome assemblies within clade RBG-13-55-18 encode a gene cluster with only *mvhD*/*hdrABC* while the other MAGs/SAGs of clades UBA1414, UBA9087, and RBG-13-55-18 do not harbor this gene cluster. The genes *hdrABC* encode a key enzyme in methanogens that is usually complexed with a [NiFe]-hydrogenase (HdrABC-MvhAGD) and functions in electron bifurcation to oxidize H_2_ coupled with the reduction of Fd and CoM-S-S-CoB (a final product of methanogenesis; heterodisulfide coenzyme M and coenzyme B) (Kaster et al., 2011; Wagner et al., 2017). The *metVF*/*mvhD*/*hdrABC* gene cluster has been observed in acetogens, such as *Moorella thermoacetica* (Mock et al., 2014) and ‘*Candidatus* Adiutrix intracellularis’ (Ikeda-Ohtsubo et al., 2016). In *Moorella thermoacetica*, this complex can reduce methylene-THF with benzyl viologen, and benzyl viologen can be reduced by NADH (Mock et al., 2014). Although the second electron acceptor remains unknown, it is likely that MetVF/MvhD/HdrABC from *Moorella thermoacetica* is capable of electron bifurcation *via* an electron-bifurcating flavin in the subunit HdrA (Mock et al., 2014). The MvhD subunit of MvhAGD contains a [2Fe-2S] cluster (Wagner et al., 2017) and could potentially function to donate electrons to HdrABC. It is possible that this *metVF*/*mvhD*/*hdrABC* cluster is regulated by the same mechanism for the catalysis of methylene-THF to methyl-THF.

The next step in the WL pathway utilizes CODH/ACS to produce acetyl-CoA. A complete or partial gene cluster for the CODH/ACS complex was identified in all genomes in the three clades except for GCA_001767735 (Rifle; clade UBA1414) and GCA_001767755 (Rifle; clade RBG-13-55-18) (Figure 2A). The CODH/ACS enzyme complex consists of five subunits, in which four share homology between *Bacteria* and *Archaea* (Adam et al., 2018; Inoue et al., 2019): *cdhA* (*acsB* in *Bacteria*; α-subunit), *cdhC* (*acsA*; β-subunit), *cdhD* (*acsD*; δ-subunit), *cdhE* (*acsC*; γ-subunit). The gene only found in *Archaea* is *cdhB* (ε-subunit) while *acsE* is unique to *Bacteria*. Notably, the Hakuba co-assembly is the only genome amongst the three clades to harbor a hybrid CODH/ACS consisting of both archaeal- (*cdhABC)* and bacterial-type (*acsCDE*) subunits (Figure 2 and Supplementary Figure S7). Although the Hakuba co-assembly did not contain the entire gene cluster for the CODH/ACS complex on one contig, the Hakuba SAG S03 had the full operon of *acsED-cdhABC-acsC*, in addition to separate gene clusters containing another *acsC*, a split *acsD*, and a split *acsE* (Figure 2B). The hybrid CODH/ACS has been observed in some putative acetogens, and several subunits have sequence similarity to subunits identified in a MAG of the candidate phylum NPL-UPA2 from the serpentinizing environment of The Cedars (Suzuki et al., 2018; Supplementary Figure S7). The hybrid CODH/ACS could be a result of horizontal transfer from *Archaea* to *Bacteria* (Adam et al., 2018; Suzuki et al., 2018). The biochemical properties of a hybrid CODH/ACS remain unknown and could provide insight into metabolisms present in serpentinizing systems and subsurface environments.

In the final steps of the WL pathway, acetyl-CoA is converted to acetate. Although none of the genomes within clades UBA1414, UBA9087, and RBG-13-55-18 harbor phosphotransacetylase (*pta*; acetyl-CoA to acetyl phosphate) or acetate kinase (*ack*; acetyl phosphate to acetate), several genome assemblies within all three clades contain homologs of acylphosphatase (*acyP*), suggesting the likely conversion of acetyl phosphate to acetate. It remains unclear whether the bacteria in all three clades can convert acetyl-CoA to acetyl phosphate, and potential genes which could replace *pta*, such as phosphotransbutyrylase (*ptb*), butyrate kinase (*buk*), or propanediol utilization protein (*pduL*) (Köpke et al., 2010; Poehlein et al., 2015), were not detected amongst any of the genomes. All genomes, except for three (Hakuba co-assembly/SAGs, BS_59E_21H_M23, and GCA_001767575), have the ADP-forming acetyl-CoA synthetase (*acd*) (Supplementary Table S10). The presence of the WL pathway and the absence of *ack* and *acd* in the Hakuba co-assembly/SAGs suggests that this bacterium likely cannot autotrophically fix CO_2_, although the genome is incomplete. It is known that autotrophic growth by acetogenesis requires the ATP generated by the action of *ack* or *acd* (Musfeldt et al., 1999; Schuchmann and Müller, 2014).

Several genomes within clades UBA1414, UBA9087, and RBG-13-55-18 harbor genes that can convert acetate to other products, such as acetyl-CoA (acetyl-CoA synthetase, *acs*), acetaldehyde (aldehyde ferredoxin oxidoreductase, *aor*), and ethanol (aldehyde-alcohol dehydrogenase, *adhE;* alcohol dehydrogenase, *adh*) (Supplementary Table S10). Acetate may be derived from several sources, including the WL pathway, the L-cysteine synthesis pathway, and the environment *via* a putative acetate transporter. However, it remains unclear whether the Hakuba actinobacterium is a bonafide acetogen as it likely cannot convert acetyl-CoA to the major end products (acetate, acetone, ethanol, and butyrate) of the WL pathway, and several genes are missing for these pathways, as mentioned above, including genes involved in acetone or butyrate synthesis (e.g., 3-hydroxybutyryl-CoA dehydrogenase and enoyl-CoA hydratase).

### Energy Conservation Mechanisms in the Hakuba Co-assembly

The Hakuba co-assembly encodes enzymes involved in energy conservation used by homoacetogens that exploit the WL pathway (Figures 3, 4 and Supplementary Table S11). In addition to hydrogenases and heterodisulfide reductase, genes coding for several subunits of the Na^+^-dependent V-type ATPase (*atpABCDEFIK*) were identified with key amino acid residues within subunit AtpK implicated in Na^+^ translocation (Mulkidjanian et al., 2008; Supplementary Figure S8). The Na^+^-dependent V-type ATPase can also translocate H^+^ (Dimroth, 1997; von Ballmoos and Dimroth, 2007). There were no genes encoding the H^+^-dependent F-type ATPase and only one subunit for the Ca^2+^/Mg^2+^-dependent P-type ATPase was identified. For the Hakuba bacterium, the translocation of Na^+^ compared to other ions (H^+^, Ca^2+^, or Mg^2+^) is likely to occur since there is about 1–1.6 mM Na^+^ in Happo #3 (Table 1), which is ten times higher than the concentration of Ca^2+^. Magnesium ions were not detected, and protons in serpentinite-hosted ecosystems are expected to be extremely low in concentration and are consumed during serpentinization, leading to alkaline pH (Okland et al., 2012).

The Hakuba co-assembly also encodes all subunits of the Rnf complex (RnfABCDEG). In model acetogens, such as *A. woodii*, the Rnf complex couples Fd oxidation to NAD^+^ reduction and Na^+^ translocation across the membrane, creating a sodium ion gradient that is subsequently utilized by the V-type ATPase for ATP synthesis (Biegel and Müller, 2010; Biegel et al., 2011; Schuchmann and Müller, 2014, 2016). The Hakuba bacterium may generate ATP via the combination of the Rnf complex and the V-type ATPase, as described above (Schuchmann and Müller, 2014, 2016). The lack of *ack* or *acd* suggests that the bacterium likely cannot conduct net ATP production *via* only the WL pathway. Thus, the bacterium would require ATP production *via* glycolysis coupled with the H^+^-translocating NADH-quinone oxidoreductase (NuoABCDGHIKLMN, NuoE, NuoF and NuoG) and dissimilatory nitrate reduction with nitrate reductase, NarGH. Reduction of nitrate *via* nitrate reductase may be coupled with the oxidation of hydrogen *via* hydrogenases, although *narG* is pseudogenized in some SAGs, as discussed below. The genome assemblies of potential acetogens with the complete WL pathway and *acd* (GCA_001768645_Rifle_CO, and GCA_001767605_Rifle_CO) also encode the V-type ATPase and Rnf complex, but not NarGH and the NADH-quinone oxidoreductase (Supplementary Table S10).

### The Hakuba Actinobacterium Is a Possible Heterotroph

**FIGURE 5:**
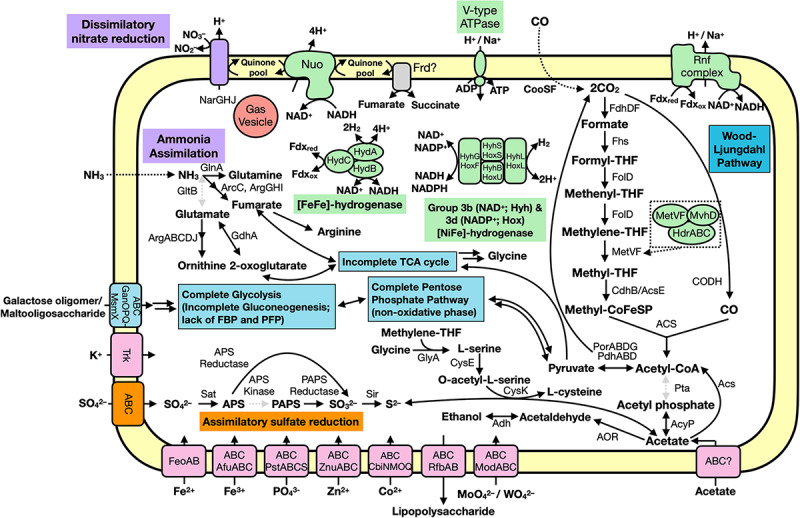
Predicted metabolic functions of the Hakuba co-assembly. Each overall feature is color coded according to metabolic function (assimilatory sulfate reduction, orange; nitrogen cycle, purple; carbon cycle, blue; transporter, pink; or energy conservation, green). Dotted, gray arrows indicate the gene is not present while the dotted box is a zoom-in of the MetVF/MvhD/HdrABC-like complex. Double arrows indicate one or more genes are involved which are not depicted. Abbreviations: ABC (ABC transporter), Acs (acetyl-CoA synthase), AcyP (acylphosphatase), Adh (alcohol dehydrogenase; as YiaY), Afu (iron(III) transport system), Arc (carbamate kinase), Arg (amino-acid N-acetyltransferase), APS (adenosine-5′-phosphosulfate), AOR (aldehyde ferredoxin oxidoreductase), Cbi (cobalt/nickel transport system), Cdh (carbon monoxide dehydrogenase), CooS (carbon monoxide dehydrogenase), CysE (serine O-acetyltransferase), CysK (cysteine synthase), Fd (ferredoxin), Fdh (formate dehydrogenase), Feo (ferrous iron transport system), Fhs [formyl-tetrahydrofolate (THF) synthase], FolD (Methylene-THF dehydrogenase), Frd (succinate dehydrogenase / fumarate reductase), Gan (maltooligosaccharide transport system), GdhA (glutamate dehydrogenase), GlnA (glutamine synthetase), GltB (glutamate synthase), GlyA (glycine hydroxymethyltransferase), Hdr (heterodisulfide reductase), Hyd (hydrogenase), Mod (molybdate transport system), MvhD (F420-non-reducing hydrogenase iron-sulfur subunit), Msm (multiple sugar transport system ATP-binding protein), NADH (nicotinamide adenine dinucleotide), Nar (nitrate reductase), Nuo (NADH-oxidoreductase), PAPS (3′-phosphoadenosine 5′-phosphosulfate reductase), Por (pyruvate ferredoxin oxidoreductase), Pst (phosphate transport system), PTS (phosphotransferase), Rfb (lipopolysaccharide transport system), Rnf (Na^+^-translocating ferredoxin:NAD^+^ oxidoreductase), Sat (ATP sulfurylase enzyme), SbtA (high affinity Na^+^-dependent bicarbonate transporter), Sir (sulfite reductase), Trk (Trk system potassium uptake protein), Znu (zinc transport system).

We further characterized the Hakuba co-assembly to ascertain the metabolic capabilities other than utilizing the WL pathway (Figure 5). The Hakuba co-assembly is capable of assimilatory sulfate reduction (see Supplementary Figure S9 and Supplementary Information) and has the complete set of genes for glycolysis (Embden-Meyerhof-Parnas pathway), converting glucose to acetyl-CoA (Supplementary Table S10). Sugars can be imported via the ABC transporter GanOPQ-MsmX and subsequently converted to glucose with genes, such as galactokinase (*galK*) and galactose-1-phosphate uridylyltransferase (*galT*). However, the Hakuba co-assembly and all SAGs lack fructose-1,6-bisphosphatase or diphosphate-dependent phosphofructokinase; therefore, it may not complete gluconeogenesis. This suggests that this bacterium cannot grow solely by carbon fixation *via* the WL pathway and is likely a heterotroph. In addition, the bacterium is likely not capable of producing major WL pathway end products from acetyl-CoA, as mentioned above, further supporting its dependence on glycolysis for energy generation. As Happo #3 is nutrient limited and organic carbon may not always be present, the Hakuba bacterium may supplement anabolic processes *via* the WL pathway.

The Hakuba co-assembly harbors all the genes for the non-oxidative pentose-phosphate pathway. The tricarboxylic acid cycle is incomplete, as seen in other known acetogens (Shin et al., 2016). Pyruvate can be reduced to malate using malate dehydrogenase, which is further converted to fumarate using fumarate hydratase. The Hakuba co-assembly contains genes for fumarate reductase catalytic, cytosolic subunits (*frdAB*), but the membrane-bound subunits are missing. The Hakuba co-assembly also contains glutamine synthetase indicating that it can potentially assimilate ammonia as a nitrogen source.

### Characterization of Nitrate Reductase and Pseudogenization of narG With Intraspecies-Variations

Nitrate reductase may confer the ability to respire nitrate for the Hakuba bacterium. Its genes, *narGHJ*, were not found in the same operon within the Hakuba co-assembly, but the intact full-length operon was identified in the Hakuba SAGs S03, S09, S34, and S42. Although the co-assembly and SAGs are missing the integral membrane subunit *narI*, which is often observed with *narGHJ* (Philippot, 2002; Cabello et al., 2004), there is at least one known denitrifying microorganism that encodes only the subunits NarGH and NarJ, *Haloarcula marismortui* (Yoshimatsu et al., 2000, 2002). It is also possible that *narI* was not sequenced, or there is a yet to be identified putative *narI*, such as observed in the secretome of *Aeropyrum pernix* K1 (Palmieri et al., 2009) but not in the genome (Kawarabayasi et al., 1999). The subunit NarH is responsible for electron transfer (Blasco et al., 1989) while NarJ is a chaperone protein necessary for assembling an active NarGH complex (Dubourdieu and DeMoss, 1992; Liu and DeMoss, 1997; Blasco et al., 1998). The subunit NarG is the catalytic subunit and can be located in either the cytoplasm or the periplasm (Richardson et al., 2001). The Hakuba co-assembly NarG has a canonical twin-arginine motif, [S/T]RR, at the N-terminal region, which is responsible for protein export to the periplasm (Martinez-Espinosa et al., 2007; Kameya et al., 2017; Supplementary Figure S10), and it phylogenetically clustered with NarG from *Hydrogenobacter thermophilus* (Supplementary Figure S11). The NarG from *H. thermophilus* was the first known bacterial NarG to be localized on the periplasmic side of the cell membrane (Kameya et al., 2017). Similar to *H. thermophilus*, the Hakuba co-assembly genome also does not contain genes for any nitrate/nitrite transporters, further supporting the periplasmic localization of NarG. After nitrate reduction, nitrite does not appear to be utilized by the Hakuba organisms. Although the Hakuba co-assembly does not encode nitrite reductase (Nir) and nitric oxide reductase (Nor), the gene neighborhood of *narGHJ* contains several Fe–S cluster-containing proteins, putative ubiquinol oxidases, and two putative *nosZ* genes. These proteins may be involved in preventing toxicity from nitrite and potentially, nitric oxide.

The gene *narG* appears to be pseudogenized by a nonsense mutation (“TGG” to “TAG”) in the Hakuba co-assembly and several SAGs (S03, S09, and S34), whereas SAGs S06, S33, and S42 have a complete *narG* sequence (Supplementary Figure S12). Examination of raw reads mapped to the region suggest that the pseudogenized *narG* was not derived from sequencing errors because the same mutation occurred at the same position on orthologous contigs among three independent samples. Moreover, this mutation was not within a homopolymeric region, which could have easily resulted in indel errors during sequencing. The potential loss of function for dissimilatory nitrate reduction in SAGs S03, S09, and S34 may arise from either functional heterogeneity amongst the individual strains of this species or an adaptation to the serpentinite-hosted environment, which has no detectable amount of nitrate or nitrite (Table 1) and may lead to the loss of NarGHJ amongst the whole population of this species. Without the utilization of nitrate, the Hakuba actinobacterium may use fumarate as an electron acceptor, although it is unclear whether the putative fumarate reductase is membrane-bound or not.

The split *narG* amongst the 10 SAGs coincide with two intraspecies-level phylotypes observed by ANI (Supplementary Table S6) and AAI (Supplementary Table S7). Based on the criteria of ANI ≥ 99% and AAI ≥ 94%, one phylotype consists of SAGs S03, S09, S34, S44, and S47 (“first phylotype”) and another phylotype consists of S25, S33, and S43 (“second phylotype”). The SAGs S06 and S42 are closer to the second phylotype, but the similarity is lower (AAI < 94%). Further examination of the genome assemblies identified 12 split genes (Supplementary Figure S13 and Supplementary Table S12), and the two phylotypes coincided with 9 out of the 12 genes. Notably, the bacterial subunit *acsE* of CODH/ACS (Figure 2B) was split only in the first phylotype (Supplementary Figure S13C). The discovery of split genes coinciding with two phylotypes clarified the strength of our approach to analyze multiple SAGs of the same species, while the co-assembly provided higher completeness and facilitated analysis of potential metabolic traits of the Hakuba actinobacterium as a species.

### Transporters, Stress Response, Motility, and RubisCO-Like Protein of the Hakuba Co-assembly

The Hakuba actinobacterium genome encodes several mechanisms that may allow it to survive in the nutrient-limited, serpentinite-hosted ecosystem of Happo #3. Several transporters are encoded by the Hakuba co-assembly, including organic carbon and inorganic ion transporters (K^+^, Fe(II/III), SO_4_^2–^, PO_4_^3–^, Zn^2+^, Co^2+^, and MoO_4_^2–^/WO_4_^2–^). In addition to the H^+^/Na^+^-dependent V-type ATPase and Na^+^/H^+^-translocating Rnf complex, the K^+^/H^+^- symporter (Trk) is needed to maintain homeostasis in an alkaline environment and to create a Na^+^/K^+^ gradient, which likely has greater electrochemical storage capacity than a proton gradient (Dibrova et al., 2015). Moreover, the Happo #3 alkaline environment contains high concentrations of K^+^ and Na^+^ compared to protons (Table 1). The genome also encodes several secondary metabolite biosynthetic gene clusters related to putative saccharide and fatty acid biosynthesis with unknown products (Supplementary Table S13). Although the function remains unknown for these pathways, secondary metabolites are known to exhibit diverse biological activities and play an important role in community interactions (Cimermancic et al., 2014).

The genome encodes a range of anti-stress and defense mechanisms, including cold and heat shock proteins (YfiA, CspA, HspR) and defense against phage infection (AbiEii toxin-antitoxin Type IV system and CRISPR/Cas system). A Cas Type IIIB operon is located near a short (99 bp) putative CRISPR sequence, which was identified at the end of the contig and could be truncated (Supplementary Figure S14). The Type III CRISPR/Cas defense mechanism produces a complex for targeted search and elimination (Wright et al., 2016). However, the corresponding virus remains unknown as the spacer within the CRISPR sequence did not match any known sequences in the CRISPRCasFinder database (Couvin et al., 2018) or the NCBI nr database. There are also three putative tyrosine-type phage integrases in the Hakuba co-assembly. One integrase is located next to a tRNA gene. In general, tRNA genes are the preferred integration site of prophages (Williams, 2002), and accordingly, this integrase region located next to a tRNA gene could be a part of a prophage. Within the gene neighborhood, there was also a Type IIG restriction-modification gene, which is one defense mechanism against ‘non-self’ DNA (Naito et al., 1995; Kobayashi, 2001).

Flagellar motility for the cells represented by the Hakuba co-assembly is unclear as there are only a few genes for biosynthesis of flagella (*fliAD*) and Type IV pili. On the other hand, the Hakuba actinobacterium may be capable of flotation using gas vesicles. The Hakuba co-assembly contains genes for several gas vesicle proteins (*gvpGK[L/F]MNOVY*) spread across three contigs, and the SAG S33 has the *gvp* genes (*gvpAGHJK*[*L/F*]*MNOV*) located on one contig (Supplementary Table S14). Gas vesicles are proteinaceous organelles, generally in the shape of a spindle or cylinder, found in both *Bacteria* and *Archaea*, and impart buoyancy to cells by allowing passive gas diffusion (e.g., O_2_, N_2_, H_2_, CO_2_, CO, and CH_4_) (Walsby, 1994; Oesterhelt, 1998; Oren et al., 2006; Coker and DasSarma, 2007; Hechler and Pfeifer, 2009; Pfeifer, 2012; Tashiro et al., 2016). The major gas vesicle protein GvpA is known to have an important role in assembling a gas vesicle while the other proteins play minor (e.g., GvpCG), regulatory (e.g., GvpDE), or unknown (e.g., GvpHI) roles (DasSarma and DasSarma, 2015). A single cell can contain several gas vesicles and likely produces gas vesicles in response to stress or environmental stimuli, such as light, oxygen concentrations, and available nutrients (Pfeifer, 2012). The Hakuba actinobacterium may synthesize gas vesicles in response to heat shock since *hsp20* (heat shock protein) is located in the same gene neighborhood as the *gvp* genes (Supplementary Table S14).

The Hakuba co-assembly encodes RubisCO (ribulose 1,5-bisphosphate carboxylase/oxygenase) Form IV protein, also known as a RubisCO-like protein (Supplementary Figure S15), which was identified in six SAGs S03, S25, S34, S42, S43, and S44. RubisCO is one of the enzymes involved in carbon fixation and is categorized into four forms. However, Form IV is known as a RubisCO-like protein because it lacks the catalytic site residue involved in the carboxylation reaction and is thought to be the ancestral form of RubisCO, arising before the great oxygenation event (Kacar et al., 2017; Erb and Zarzycki, 2018). Some RubisCO-like proteins are known to be involved in the methionine salvage pathway (Ashida, 2003; Erb et al., 2012) and the degradation of four carbon sugar acids (Zhang et al., 2016). The full metabolic range of RubisCO-like proteins remains unknown, although most are likely isomerases and/or epimerases (Erb and Zarzycki, 2018). In the Hakuba actinobacterium, the RubisCO-like protein may function as an epimerase acting on sugars, as the gene neighborhood of the RubisCO-like protein harbors genes with an epimerase conserved domain (cd09023) and a sugar substrate binding site (DeoR C-terminal sensor domain, PF00455). Closely related RubisCO-like proteins to the Hakuba co-assembly, as determined by BLASTp searches, include those from the Candidate Phyla Radiation, *Spirochaetes*, *Planctomycetes*, and the model acetogen *Moorella thermoacetica*. The functions of RubisCO-like proteins in these genomes are also unknown.

## Conclusion

Terrestrial serpentinite-hosted ecosystems are important modern-day analogs of early Earth and can also provide insights into processes that may have supported life at that time. Here, we present a genomic characterization of a dominant member in the Hakuba Happo hot spring ecosystem that belongs to the early-branching actinobacterial clade UBA1414. Single-cell genomics revealed that the bacterium utilizes the WL pathway for converting CO_2_ to acetyl-CoA and could be represented by two phylotypes within a single species. We also identified related genome assemblies that encode the WL pathway; these bacteria are the first known to encode the WL pathway within the *Actinobacteria* phylum. Within Happo #3, examination of other dominant members, such as “Parcubacteria” and *Nitrospirae*, will further reveal the characteristics of this ecosystem. On the basis of the single-cell genome sequences, we propose a novel order ‘*Candidatus* Hakubanellales’ and novel family ‘*Candidatus* Hakubanellaceae.’ We propose to name this bacterium ‘*Candidatus* Hakubanella thermoalkaliphilus’ as described below.

### Description of ‘*Candidatus* Hakubanella’ gen. nov

*Hakubanella* [ha.ku.ba.nel’la, N.L. fem. dim. n. *Hakubanella* of Hakuba Happo, a serpentinite-hosted environment located in Nagano (Japan) from where the single-cell genome assemblies were obtained. The type species is ‘*Candidatus* Hakubanella thermoalkaliphilus’ with the single-cell genome assemblies as the type material.

### Description of ‘*Candidatus* Hakubanella thermoalkaliphilus’ sp. nov

*Hakubanella thermoalkaliphilus* (ther.mo.al.ka.liphil.us, Gr. adj. *thermos* hot; N.L. n. *alkali* from Arabic *al*-*qaliy* the ashes of saltwort; Gr. adj. *philos* friend, loving; N.L. adj. *alkaliphilus* liking alkaline environments). The genome of the bacterium was discovered in Hakuba Happo hot springs, where temperatures reach about 50°C and pH∼11. Based on genome analysis, the bacterium is anaerobic and possesses glycolysis for energy harvesting and the WL pathway for anabolic processes. The bacteria can potentially utilize sugars and accommodate two phylotypes: one is capable of dissimilatory nitrate reduction and another has lost the activity. The bacteria probably cannot grow autotrophically. The assignment is based on single-copy taxonomic marker genes.

## Data Availability Statement

The 16S rRNA amplicon sequences (DRR198702–DRR198704 under DRA009263), the raw fastq files (DRR198705–DRR198714 under DRA009264), and cleaned assemblies for SAGs (BLRU01000000–BLRZ01000000, BLSA01000000–BLSD01000000) and the co-assembly (BLSE01000000) were deposited into DDBJ under BioProject accession no. PRJDB8357. The gas vesicle hidden Markov model database is available on GitHub at https://github.com/Arkadiy-Garber/MagicCave/tree/master/hmms/gas.

## Author Contributions

NM, KK, and YH designed the study. NM contributed to the field sampling and the collection, sequencing, and bioinformatics analyses of the single-cell genomes. MK and YH contributed to the sequencing and bioinformatics analyses. EB and DC contributed to analysis of hydrogenases. NM, MK, EB, DC, SM, KN, KK, and YH wrote the manuscript.

## Conflict of Interest

The authors declare that the research was conducted in the absence of any commercial or financial relationships that could be construed as a potential conflict of interest.

## Footnotes

1 https://www.bioinformatics.babraham.ac.uk/projects/fastqc/

2 https://www.bioinformatics.babraham.ac.uk/projects/trim_galore/

3 http://hmmer.org

4 https://github.com/tseemann/barrnap/blob/master/README.md

5 https://jgi.doe.gov/data-and-tools/bbtools/bb-tools-user-guide/bbnorm-guide/

6 https://github.com/widdowquinn/pyani

7 https://github.com/AstrobioMike/GToTree/tree/master/hmm_sets

8 http://tree.bio.ed.ac.uk/software/figtree/
