# Supplementary material for: Single-Cell Genomics of Novel Actinobacteria With the Wood–Ljungdahl Pathway Discovered in a Serpentinizing System
Source: Front Microbiol. 2020 Jun 9;11:1031. doi: 10.3389/fmicb.2020.01031 (PMC7325909; doi:10.3389/fmicb.2020.01031)
Supplement: Supplementary file 1 [file Data_Sheet_1.pdf]

Supplementary Information

Single-Cell Genomics of Novel Actinobacteria with the Wood-Ljungdahl Pathway Discovered in a Serpentinizing System

Nancy Merino[a,b,c*], Mikihiko Kawai[d,e], Eric S. Boyd[f], Daniel R. Colman[f], Shawn E. McGlynn[a,g,h], Ken Nealson[b], Ken Kurokawa[a,i], Yuichi Hongoh[a,d*]

[a] Earth-Life Science Institute, Tokyo Institute of Technology, Tokyo, Japan
[b] Department of Earth Sciences, University of Southern California, Los Angeles, CA, USA
[c] Biosciences and Biotechnology Division, Lawrence Livermore National Lab, Livermore, CA, USA
[d] School of Life Science and Technology, Tokyo Institute of Technology, Tokyo, Japan
[e] Graduate School of Human and Environmental Studies, Kyoto University, Kyoto, Japan
[f] Department of Microbiology and Immunology, Montana State University, Bozeman, MT, USA
[g] Biofunctional Catalyst Research Team, RIKEN Center for Sustainable Resource Science (CSRS), Saitama, Japan
[h] Blue Marble Space Institute of Science, Seattle, WA, USA
[i] Department of Informatics, National Institute of Genetics, Shizuoka, Japan

Running Title: Genome of Novel Actinobacteria

***Correspondence:**
Nancy Merino
nmerino@elsi.jp

Yuichi Hongoh
yhongo@bio.titech.ac.jp

Materials and Methods Supplementary Information

Measurement of Ions and Organic Acids

Water was collected and stored at -4ºC for ion (Ca^{2+}, NH_4^+, Mg^{2+}, Cl^-, SO_4^{2-}, NO_2^-, NO_3^-, HCO_3^-, and HPO_4^-) and organic acid (formate, acetate, propionate, pyruvate, and lactate) analyses using ion chromatography (Shodex Ion Chromatogram SI-90G, Japan) with an IC-C4 Shim-pack column (150 mm length, 4.6 mm ID, carboxylic polymethacrylate) connected to a guard column. Nucleosides and nucleobases were analyzed by LCMS in the ESI-positive mode and reversed phase UPLC (column: ACQUITY UPLC® BEH C18 1.7 µm). Elution conditions included: 0.3 ml/min at a constant flow rate for 0–4 min, then 99% A (A: 0.1% TFA) and 1% B (B: acetonitrile), followed by a linear gradient for 7 min to 50% A and 50% B and then, 7.8–8.3 min of 10% A and 90% B. Finally, a linear gradient was conducted for 8.5 min until reaching the initial conditions. Analysis of amino acids was conducted by a liquid chromatograph (ICA-2000; TOA DKK) equipped with a UV detector (UV-2075; Jasco) operated at a wavelength of 200 nm. For chromatography, a reverse-phase type column (Hydrosphere C18; YMC) (Ohara et al., 2007) was used at 37ºC. A 10 mM ($C_6H_{13}SO_3Na$ solution with pH 2.5 was used as an eluent (adjusted by H_3PO_4) (Bujdák and Rode, 1999), at a flow rate of 1.0 ml/min. Measurement errors for concentrations of Gly, GlyGly, and DKP were estimated to be less than 3.0%.

FACS Parameters

Several FACS parameters were modified to prevent sorting of particles not representative of the Hakuba Happo microbial community (FSC Threshold 750, Area Scaling 0.82, Blue Scaling 0.96, SSC Threshold 200), and the microbial community was selected based on four parameters to prevent doublets: FSC vs SSC, FSC-H vs FSC-A, SSC-H vs SSC-A, and FITC-A vs FITC-H. Three different phase masks (PM) were used to sort the samples (16, 20, and 29) in the "single cell" mode and the number of cells sorted was confirmed by inverted fluorescence microscopy to ensure single sorted cells. Only one phase mask was used per 96-well plate.

Single Cell Lysis and Whole Genome Amplification

Each plate was thawed from -80ºC and kept on ice during cell lysis and WGA. Three different lysing conditions were tested for each plate: 1) 10 mM Tris (Tris), 2) 50 U/µL Ready-Lyse™ Lysozyme Solution (Lys) (Epicentre, WI, U.S.) (Goudeau et al., 2014), 3) 50 U/µL Lysozyme with Mid-Alkaline Buffer (Lys+MidAlk), and 4) 10 mM Tris with Mid-Alkaline Buffer (Tris+MidAlk). Mid-Alkaline Buffer contained 18 mM KOH, 0.45 mM EDTA, and 4.5 mM DTT (from REPLI-g kit). The following solutions were UV-treated in a UV Crosslinker (254nm, 100V, 8W; UVP, LLC) for 60 min: Mid-Alkaline Buffer (without DTT), Neutralization Buffer, STOP buffer, 10 mM Tris pH 8, and nuclease-free H_2O. Initially, each well was incubated with 1 µL Tris (Tris and Tris+MidAlk) or 1 µL Lysozyme (Lys and Lys+MidAlk) for 15 min at 25ºC. Afterwards, each well was incubated with 0.75 µL of Buffer DB (Tris and Lys) or Mid-Alkaline Buffer (Lys+MidAlk) for 10 min at 25ºC. Cell lysis was stopped using 0.75 µL STOP buffer (Tris and Lys) or Neutralization Buffer (Lys+MidAlk and Tris+MidAlk). Neutralization Buffer stock contained 20 mM HCl and 30 mM Tris-HCl. WGA was performed using (per 10 µL reaction) 2.25

μL H_2O, 7.25 μL Reaction Buffer, and 0.5 μL Phi29 polymerase. Each plate was centrifuged for 1000 g for 1 min at 4ºC, followed by incubation at 30ºC for 14 h, 65ºC for 10 min, and 4ºC infinite in a PCR instrument with cover heated to 70ºC.

Gas Vesicle HMM database

Gas vesicle proteins were annotated using a manually curated hidden Markov model database. The database was created using a similar method as FeGenie (Garber et al., 2019). Briefly, gas vesicle protein sequences were obtained from the UniProt database (Bateman et al., 2017) and used as queries against the NCBI RefSeq database (Release 93) in a BLASTp (v2.3.0) (Camacho et al., 2009) search. By using a minimum amino acid identity cutoff of 35% (Rost, 1999) over at least 70% of the query length, the additional sequences expanded the diversity of each collected gas vesicle protein. Subsequently, the sequences were de-replicated and overrepresented protein sequences were removed with MMSeqs2 (Steinegger and Söding, 2017). MAFFT v7.055b (Katoh et al., 2002) was used to align each set of sequences and trimAI v1.4.rev15 (Capella-Gutierrez et al., 2009) was used to remove gaps. Manual curation was done before generating hidden Markov models using HMMER (Mistry et al., 2013). This database was then used to annotate gas vesicle proteins in the co-assembled genome and SAGs. Since no bitscore cutoff was used, the annotation was manually checked and corrected with a BLASTp search against the NCBI non-redundant (nr) database.

Split Members of an Orthologous Group of Genes at the Domain Level

Orthologous groups (OGs) were generated by the DomClust program (Uchiyama, 2006). Default parameters were used except for the following: -ai1.0 -ao1.0 -p0.0 -V0.2. Parameters of -ai1.0 -ao1.0 were used to obtain a phylogenetic profile constructed for each OG using the number of domains for each gene (Kawai et al., 2011, 2014). Other parameters included were -P10 (distance of PAM10 to select sequence pairs for the input to obtain only very similar sequences as a member of an OG), -p0.0 (to cut a tree into sub-trees whenever genes of the same organism are found in sub-trees and to avoid merging paralogous groups, which are complementary and may occur because of the incomplete nature of SAGs), -V0.2 (to split clusters into domains of shorter length when a complementary pair of members of short length are included in a single cluster and can interfere with the analysis of the number of paralogs). For SAGs, there are many short, truncated genes generated through multiple displacement amplification (MDA). Following DomClust, several post-processing steps were utilized to decrease potential false positives resulting from MDA. Only members with full-length genes, i.e. genes non-truncated at the ends of a contig, were considered among the members of each OG. The gene neighborhood was also examined as short contigs (a few kb) may result in short genes and the orientation and order of genes within the contig may be different from ortholog members in genomes. Such spurious genes of different order may have been generated by the mechanism of chimera formation during MDA (Lasken and Stockwell, 2007). To reduce such spurious cases, only members with at least 3 genes

at both flanking regions (≤ 10kb at both sides) were considered. Gene neighborhoods were examined using RECOG system (Uchiyama, 2017).

Results and Discussion Supplementary Information

The Hakuba Co-assembly Encodes Assimilatory Sulfate Reduction

The Hakuba co-assembly encodes genes for assimilatory sulfate reduction, converting sulfate to sulfide. Sulfate is likely transported into the cell by a putative sulfate ABC transporter and is subsequently activated by an ATP sulfurylase enzyme (Sat) to adenosine-5'-phosphosulfate (APS). The Hakuba co-assembly can then directly reduce APS to sulfite with APS reductase. A homolog to APS reductase was also identified in the Hakuba co-assembly: PAPS (3'-phosphoadenosine 5'-phosphosulfate) reductase. Genomes with both APS and PAPS reductase have been observed previously, such as in methanotrophs (Yu et al., 2018). Although APS and PAPS reductase utilize the same catalytic mechanism (Carroll et al., 2005), the substrate of PAPS reductase in the Hakuba co-assembly is unknown, especially when considering that PAPS is likely not produced. Indeed, the PAPS reductase from the Hakuba co-assembly phylogenetically clustered with other PAPS reductases with unknown substrate (**Figure S9**).

Split Genes Among the 10 Hakuba SAGs

The split status of genes among the 10 Hakuba SAGs were examined by generating OGs at the domain level. This revealed 179 OGs at the domain level that constitute 105 genes that are split into shorter, multiple genes in some of the 10 SAGs (**Table S12**). The non-split genes consisted of longer genes with two or more domains per gene compared to the split genes. To reduce the possibility that the split genes emerged from experimental errors, such as errors during MDA or sequencing, 12 cases among the 105 are marked in **Table S12**, in which both types of genes, split type and non-split type, were detected among more than 1 genome. For the 12 cases, the distribution pattern of split/non-split members among the 10 SAGs of 9 cases coincided with the separation of SAGs into two major intraspecies-level phylotypes observed by ANI (**Table S6**) and AAI (**Table S7**): one phylotype includes S03, S09, S34, S44, and S47 and another phylotype includes S25, S33, and S43 (S06 and S42 have weaker similarity). The gene neighborhood of the following genes, marked in the table, are illustrated in **Figure S13** (A. *narG*, B. *nox1/hcaD,* C. *acsE*, D. *cooS*, E. gas vesicle protein *gvpF*).

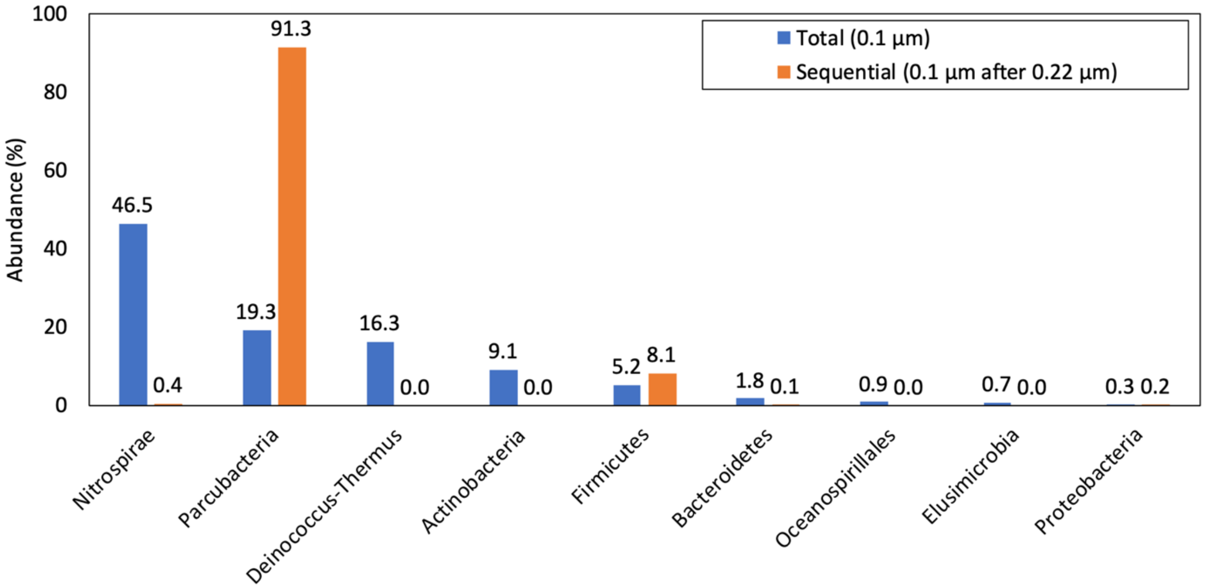

Figure S1. Bacterial community composition of Hakuba Happo well #3. The microbial community has low diversity and consists of six major phyla (>1% abundance). DNA was extracted from the 0.1 µm filter fraction of the "Total" (water filtered through 0.1 µm filter) and "Sequential" samples (water filtered through 0.22 µm sterivex, followed by 0.1 µm filter). The "Sequential" sample represents only the 0.1 µm bacterial community fraction. The Illumina MiSeq platform was used to obtain the V3–V4 region 16S rRNA gene amplicon sequences, and the reads were analyzed using DADA2 (Callahan et al., 2016).

Figure S2A – Anvi'o profiles

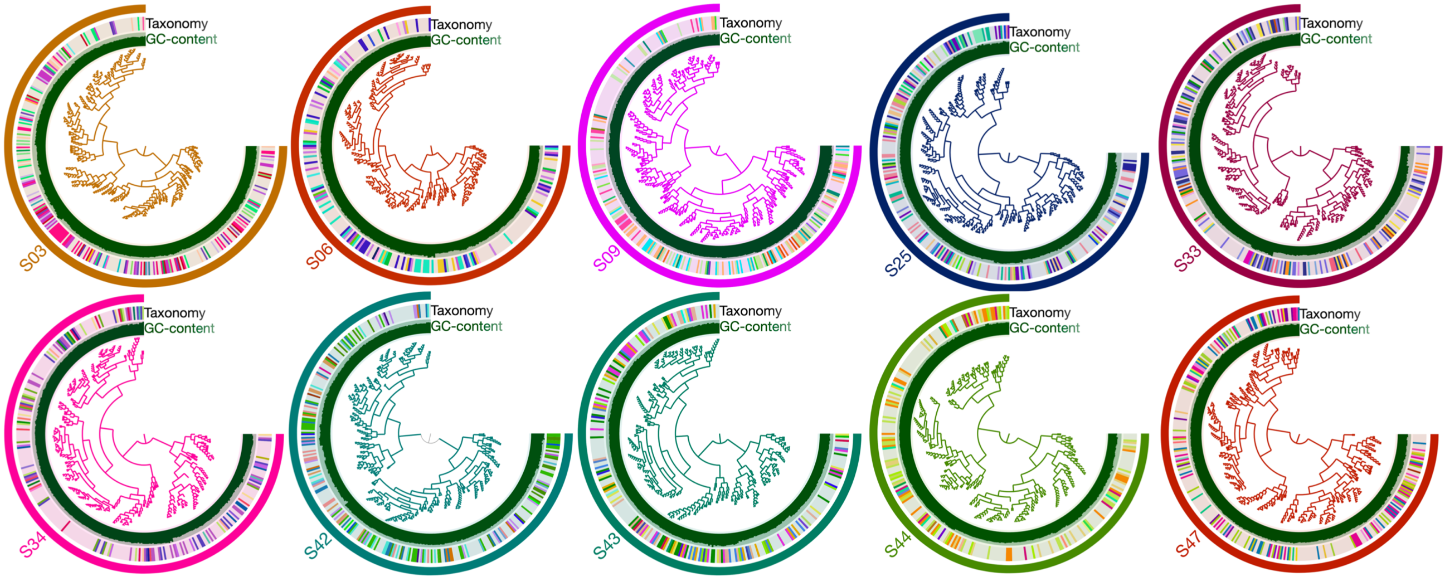

Figure S2B – ACDC Result

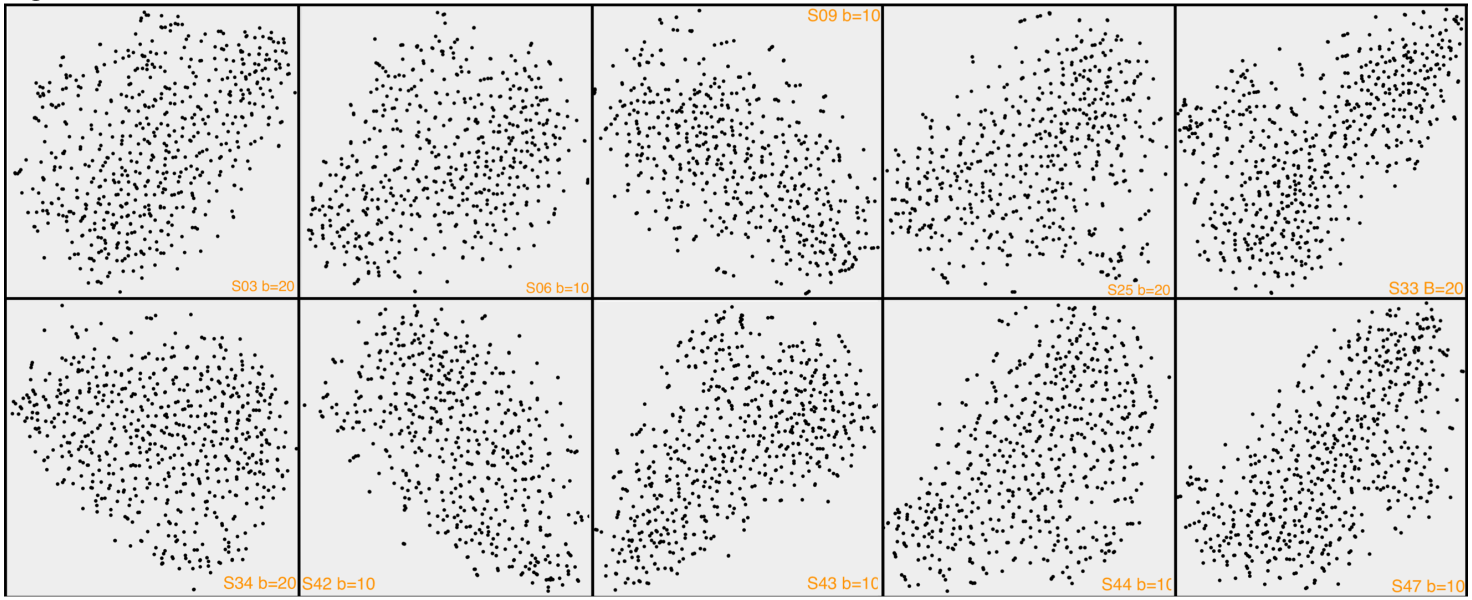

Figure S2C – ACDC Result of Co-assembly

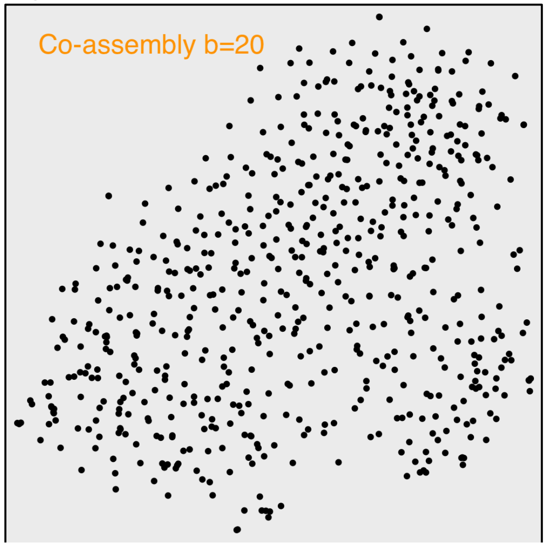

Figure S2. Taxonomy, GC content, and clustering of contigs from the 10 SAGs. (A) Anvi'o v5.3 workflow (Eren et al., 2015) was used to cluster the contigs by sequence composition (k-mer frequency = 4) and Kaiju v1.5.0 (Menzel et al., 2016) was used for taxonomic classification. Taxonomic classification is color coded by phylum level, according to the Anvi'o automatic coloring when using anvi-interactive. Since the SAGs could not be viewed together using anvi-interactive, the color code scheme is different for the contig taxonomic classification of each SAG. However, the variation in color for each SAG demonstrates the inconsistency in the taxonomic classification of contigs. GC content is shown as the inner circle in green. **(B and C)** ACDC (Lux et al., 2016) was used for contamination screening of SAGS **(B)** and Co-assembly **(C)** with default values and bootstrap (b) of either 10 or 20 and BH-SNE dimension reduction was plotted.

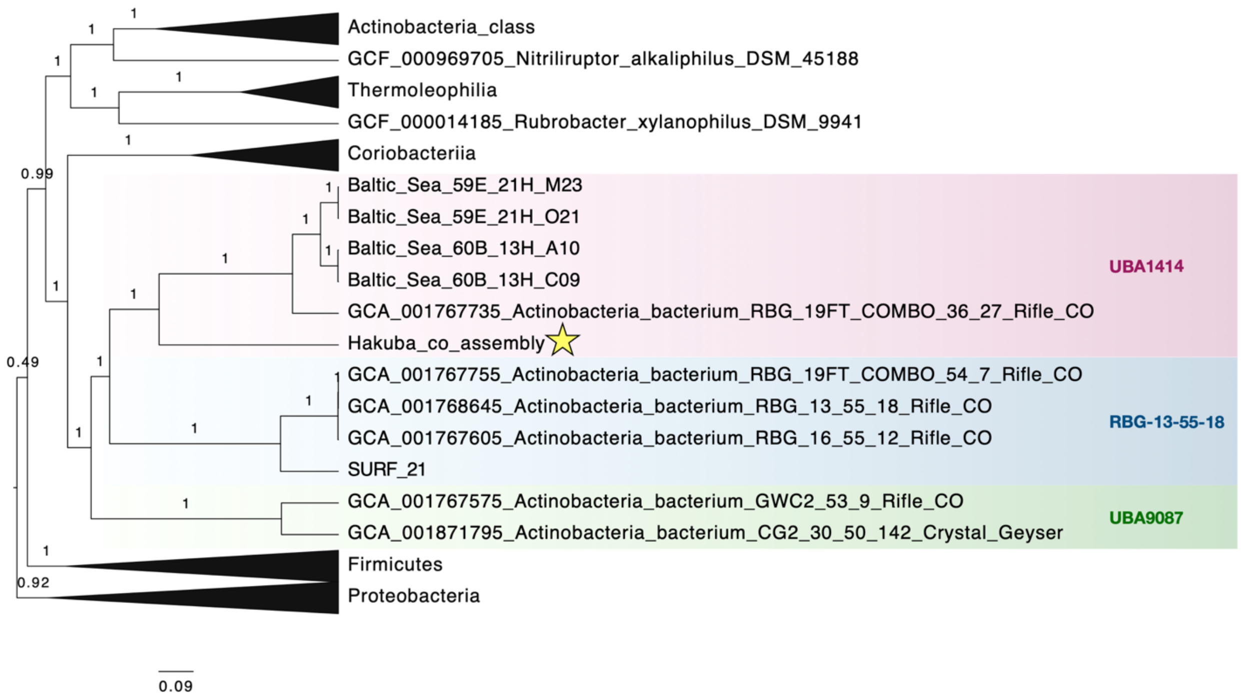

Figure S3. Bayesian phylogenetic tree of clades UBA1414, RBG-13-55-18, and UBA9087. Members of *Firmicutes* and *Proteobacteria* were used as outgroups. The genome sequences used to create this tree are listed in Table S3 and the Hakuba co-assembly is denoted by a star. Each branch was evaluated with the Bayesian posterior probability.

```
Coassembly_2912                                   1 MR-VTSVVCPFCGALCDDIEIEVEGDVIKGVKRGCALSKSFFLHAEDLSHPLVEGLEVE 58
S03_1304                                          1 MR-VTSVVCPFCGALCDDIEIEVEGDVIKGVKRGCALSKSFFLHAEDLSHPLVEGLEVE 58
S09_1465                                          1 ---------------CDDIEIEVEGDVIKGVKRGCALSKSFFLHAEDLSHPLVEGLEVE 44
S47_1148                                          1 MR-VTSVVCPFCGALCDDIEIEVEGDVIKGVKRGCALSKSFFLHAEDLSHPLVEGLEVE 58
S34_1370                                          1 MR-VTSVVCPFCGALCDDIEIEVEGDVIKGVKRGCALSKSFFLHAEDLSHPLVEGLEVE 58
O74032_FwdB_Methanothermobacter_wolfeii           1 MEYVKNVVCPFCGTLCDDIICKVEGNEIVGTINACRIGHSKFVHAERYKKPLIEFVEVS 59
ACS39602_1_FhcB_Methylorubrum_extorquens_AM1      1 MA------------------------------------------------AWVKGGAAD 11

Coassembly_2912                                  59 LEEAVEEATRILARADYPLIYGLSCTTIEAQRKAMELADLLGANIDSTSSICHGPTGIA 117
S03_1304                                         59 LEEAVEEATRILARADYPLIYGLSCTTIEAQRKAMELADLLGANIDSTSSICHGPTGIA 117
S09_1465                                         45 LEEAVEEATRILARADYPLIYGLSCTTIEAQRKAMELADLLGANIDSTSSICHGPTGIA 103
S47_1148                                         59 LEEAVEEATRILARADYPLIYGLSCTTIEAQRKAMELADLLGANIDSTSSICHGPTGIA 117
S34_1370                                         59 LEEAVEEATRILARADYPLIYGLSCTTIEAQRKAMELADLLGANIDSTSSICHGPTGIA 117
O74032_FwdB_Methanothermobacter_wolfeii          60 YDEAIDKAAKILAESKRPLMYGWSCTECEAQAVGVELAEEAGAVIDNTASVCHGPSVLA 118
ACS39602_1_FhcB_Methylorubrum_extorquens_AM1     12 VDAAVEAAADLLAASRVPVLAGLS-AEVSALRAAYRLAETLGASLDPVSGPSVYAELGA 69

Coassembly_2912                                 118 MQMVGVATCTLGEIKNRADLLVFWGCNPAESHPRHFSRYSALAKGLLTPRGRKDRTVVV 176
S03_1304                                        118 MQMVGVATCTLGEIKNRADLLVFWGCNPAESHPRHFSRYSALAKGLLTPRGRKDRTVVV 176
S09_1465                                        104 MQMVGVATCTLGEIKNRADLLVFWGCNPAESHPRHFSRYSALAKGLLTPRGRKDRTVVV 162
S47_1148                                        118 MQMVGVATCTLGEIKNRADLLVFWGCNPAESHPRHFSRYSALAKGLLTPRGRKDRTVVV 176
S34_1370                                        118 MQMVGVATCTLGEIKNRADLLVFWGCNPAESHPRHFSRYSALAKGLLTPRGRKDRTVVV 176
O74032_FwdB_Methanothermobacter_wolfeii         119 LQDVDYPICTFGEVKNRADVVVYWGCNPMHAHPRHMSR-NVFARGFFRERGRSDRTLIV 176
ACS39602_1_FhcB_Methylorubrum_extorquens_AM1     70 LSAGGAMSTTRAETIGRADVILIVGNRPAAAAPSR-GRAAGSERALLSLGGPQNGAIRH 127

Coassembly_2912                                 177 VDVRPSASSHTADIFLQINPNGDFECLWVLRALLKGEKVDLEEVSGIAVEELRELSERM 235
S03_1304                                        177 VDVRPSASSHTADIFLQINPNGDFECLWVLRALLKGEKVDLEEVSGIAVEELRELSERM 235
S09_1465                                        163 VDVRPSASSHTADIFLQINPNGDFECLWVLRALLKGEKVDLEEVSGIAVEELRELSERM 221
S47_1148                                        177 VDVRPSASSHTADIFLQINPNGDFECLWVLRALLKGEKVDLEEVSGIAVEELRELSERM 235
S34_1370                                        177 VDVRPSASSHTADIFLQINPNGDFECLWVLRALLEGEKVDLEEVSGIAVEELRELSERM 235
O74032_FwdB_Methanothermobacter_wolfeii         177 VDPRKTDSAKLADIHLQLDFDRDYELLDAMRACLLGHEILYDEVAGVPREQIEEAVEVL 235
ACS39602_1_FhcB_Methylorubrum_extorquens_AM1    128 VAYAADAGGLTIS-------------LGHLRAFAKGH------LAGEAA--FADLAKRL 165

Coassembly_2912                                 236 KGARFGVLLFG------MGLTMTRGRHLNVLAALTLTRDLNQFSKFAAV----PMRGHG 284
S03_1304                                        236 KGARFGVLLFG------MGLTMTRGRHLNVLAALTLTRDLNQFSKFAAV----PMRGHG 284
S09_1465                                        222 KGARFGVLLFG------MGLTMTRGRHLNVLAALTLTRDLNQFSKFAAV----PMRGHG 270
S47_1148                                        236 KGARFGVLLFG------MGLTMTRGRHLNVLAALTLTRDLNQFSKFAAV----PMRGHG 284
S34_1370                                        236 KGARFGVLLFG------MGLTMTRGRHLNVLAALTLTRDLNQFSKFAAV----PMRGHG 284
O74032_FwdB_Methanothermobacter_wolfeii         236 KNAQFGILFFG------MGITHSRGKHRNIDTAIMMVQDLNDYPRWTLI----PMRGHY 284
ACS39602_1_FhcB_Methylorubrum_extorquens_AM1    166 FAAQYGVIVYDPEEVGELGAEMLQG----------LIRDLNESTRFFALTLADPFQGRA 214

Coassembly_2912                                 285 NVSGIDALSAWQTGYPFGVNFSRGYPQYNPGEFTTVDILARKEVDAALIIASDPYANLP 343
S03_1304                                        285 NVSGIDALSAWQTGYPFGVNFSRGYPQYNPGEFTTVDILARKEVDAALIIASDPYANLP 343
S09_1465                                        271 NVSGIDALSAWQTGYPFGVNFSRGYPQYNPGEFTTVDILARKEVDAALIIASDPYANLP 329
S47_1148                                        285 NVSGIDALSAWQTGYPFGVNFSRGYPQYNPGEFTTVDILARKEVDAALIIASDPYANLP 343
S34_1370                                        285 NVSGIDALSAWQTGYPFGVNFSRGYPQYNPGEFTTVDILARKEVDAALIIASDPYANLP 343
O74032_FwdB_Methanothermobacter_wolfeii         285 NVTGFNQVCTWESGYPYCVDFSGGEPRYNPGETGANDLLQNREADAMMVIASDPGAHFP 343
ACS39602_1_FhcB_Methylorubrum_extorquens_AM1    215 AV----QLSAWTTGQAPRVGFGRHQPEHDSWRFDSARQIAAGEADAALWLAS-----LP 264

Coassembly_2912                                 344 RRAAQRLEEIPTIVMDPKRSKTA--QIARVVIPTAISGISAEGTAYRMDDVPLRLKRLI 400
S03_1304                                        344 RRAAQRLEEIPTIVMDPKRSKTA--QIARVVIPTAISGISAEGTAYRMDDVPLRLKRLI 400
S09_1465                                        330 RRAAQRLEEIPTIVMDPKRSKTA--QIARVVIPTAISGISAEGTAYRMDDVPLRLKRLI 386
S47_1148                                        344 RRAAQRLEEIPTIVMDPKRSKTA--QIARVVIPTAISGISAEGTAYRMDDVPLRLKRLI 400
S34_1370                                        344 RRAAQRLEEIPTIVMDPKRSKTA--QIARVVIPTAISGISAEGTAYRMDDVPLRLKRLI 400
O74032_FwdB_Methanothermobacter_wolfeii         344 QRALERMAEIPVIAIEPHRTPTT--EMADIIIPPAIVGMEAEGTAYRMEGVPIRMKKVV 400
ACS39602_1_FhcB_Methylorubrum_extorquens_AM1    265 APRPAWLGSLPTIAIVGEGSQEAAGETAEVVITVGVPGQSVGGALWN-DRRGVIAYAEA 322

Coassembly_2912                                 401 SSP--YPPDHEVLDEIIRRVKKCLGYQEERSMTLSMA 435
S03_1304                                        401 SSP--YPPDHEVLDEIIRRVKKCLGYQEERSMTLSMA 435
S09_1465                                        387 SSP--YPPDHEVLDEIIRRVKKCLGYQEERSMTLSM- 420
S47_1148                                        401 SSP--YPPDHEVLDEIIRRVKKCLGYQEERSMTLSMA 435
S34_1370                                        401 SSP--YPPDHEVLDEIIRRVKKCLGYQEERSMTLSMA 435
O74032_FwdB_Methanothermobacter_wolfeii         401 DSA--SSQTGRSLRDSLRR------------------ 417
ACS39602_1_FhcB_Methylorubrum_extorquens_AM1    323 SDPAKTPAETETAAGVLTRIRDRLIEKGVSC------ 353
```

Figure S4. Multiple sequence alignment of *fwdB* from the Hakuba genome assemblies and *Methanothermobacter wolfeii* against the *fhcB* from *Methylorubrum extorquens*. The Fwd complex is the key enzyme involved in the first step of CO_2 reduction in methanogenesis while the Fhc complex converts formyl-H_4MPT (tetrahydromethanopterin) to formate (Pomper et al., 2002; Adam et al., 2019; Hemmann et al., 2019). The subunits of the Fwd and Fhc complex share homology. However, FwdB contains the tungstopterin active site, which is not present in FhcB (Hemmann et al., 2019). FhcB is missing the N-terminal domain which contains the [4Fe-4S] cluster (green highlight in *Methanothermobacter wolfeii*). The catalytic Cys118 is also replaced by a Ser62 in FhcB (highlighted green column), and the amino acid sequence of FhcB also contains loops that would prevent a tungstopterin (red highlights).

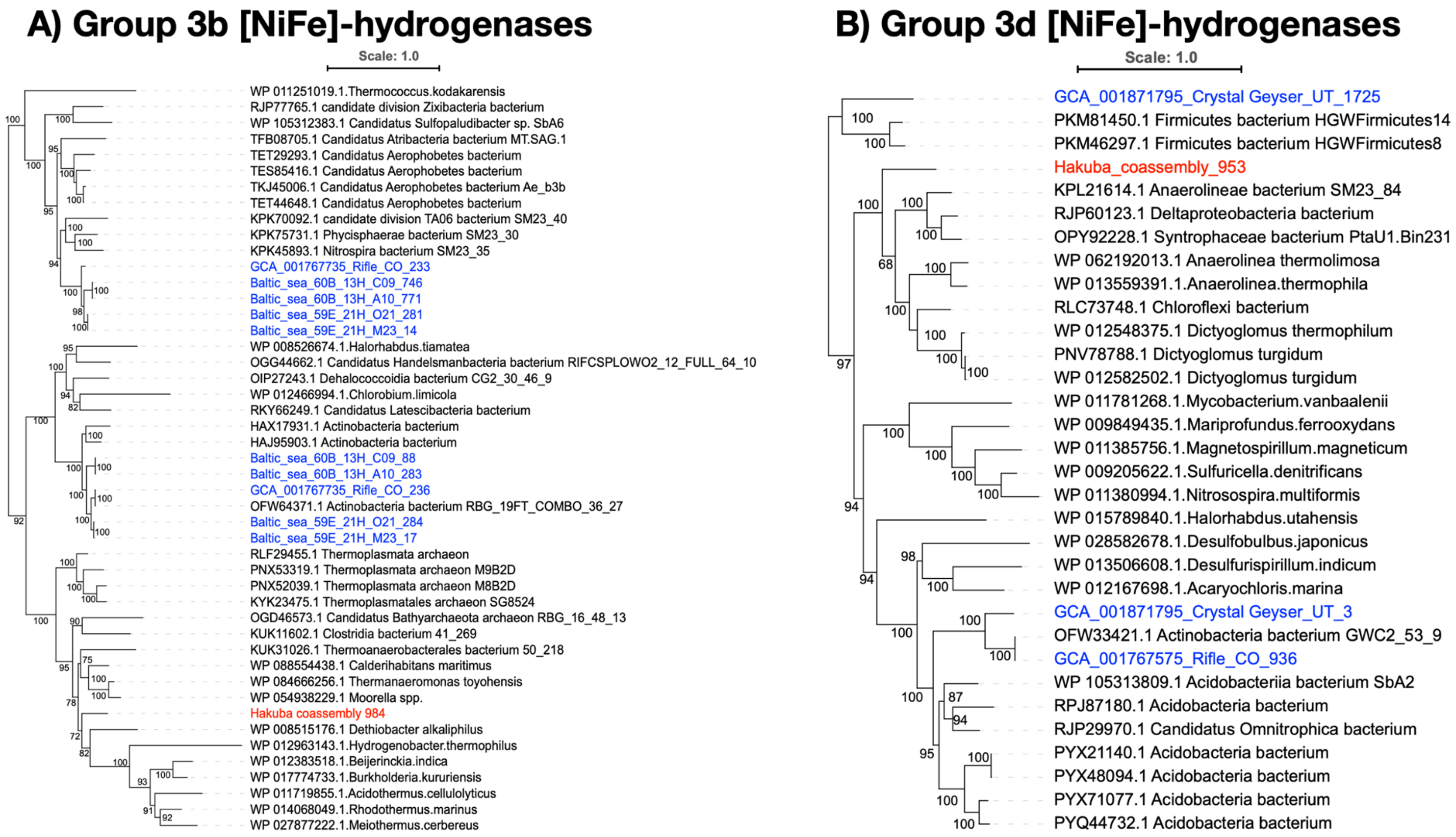

Figure S5. Maximum likelihood phylogenetic tree of Group 3b and 3d [NiFe]-hydrogenases. The Hakuba co-assembly (red text) and other genomes within clade UBA1414, UBA9087, and RBG-13-55-18 (blue text) encode for group 3b and/or 3d [NiFe]-hydrogenases. The IQtree maximum likelihood algorithm with the LG+G amino acid substitution model was used with 1000 bootstraps to evaluate node support. Sequences from the representatives of the respective [NiFe]-hydrogenase isoform groups and close representatives to the query sequences (NCBI database) were used.

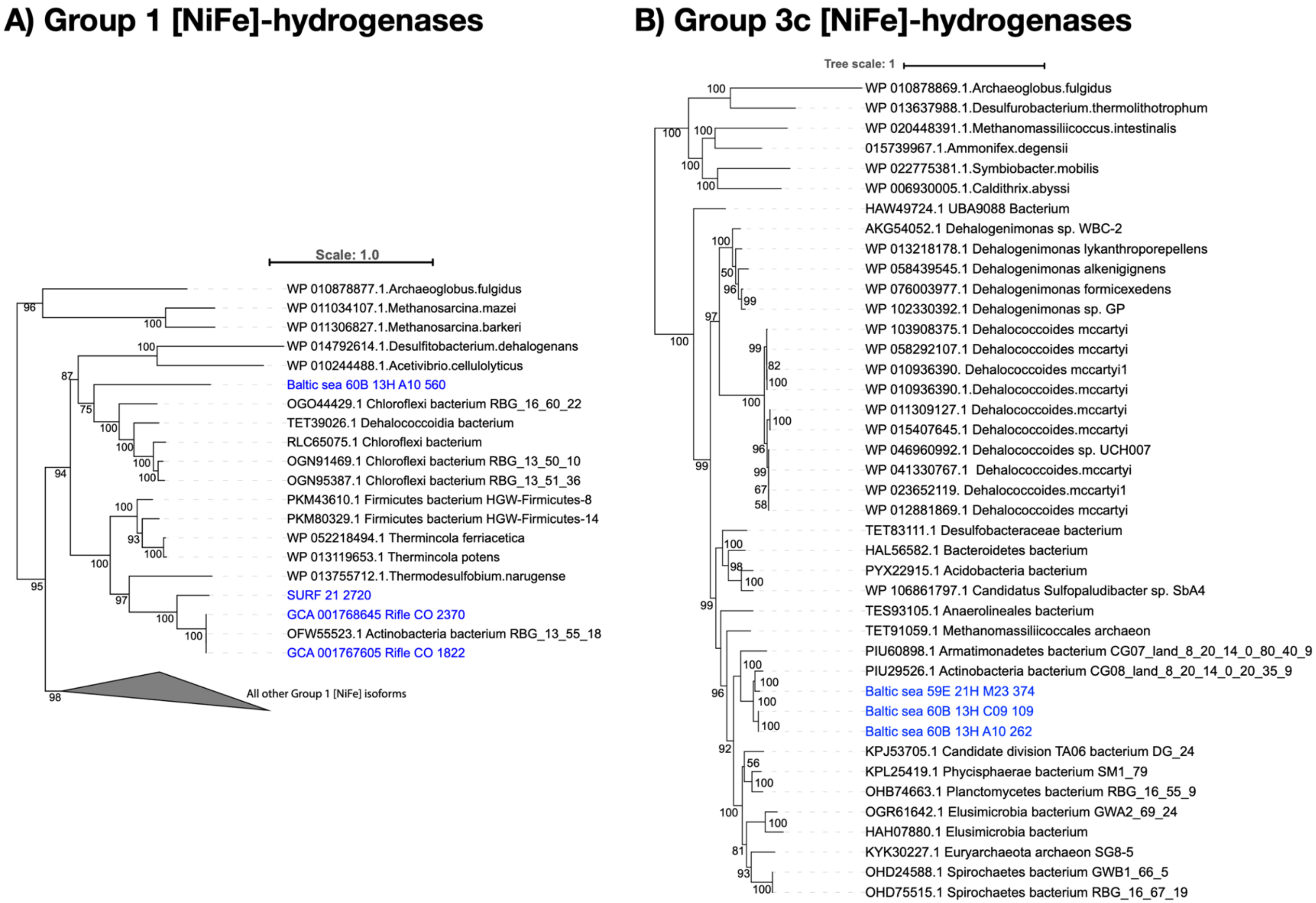

Figure S6. Maximum likelihood phylogenetic tree of Group 1 and 3c [NiFe]-hydrogenases. The Hakuba co-assembly did not encode for group 1 and 3c [NiFe]-hydrogenases, although several other genomes within clade UBA1414, UBA9087, and RBG-13-55-18 (blue text) harbored the respective genes. The Group 3c [NiFe]-hydrogenase clade shown represented an outgroup to prototypical methanogen 3c hydrogenases, which are not shown for brevity. The IQtree maximum likelihood algorithm with the LG+G amino acid substitution model was used with 1000 bootstraps to evaluate node support. Sequences from the representatives of the respective [NiFe]-hydrogenase isoform groups and close representatives to the query sequences (NCBI database) were used.

Figure S7A – CdhA/AcsB

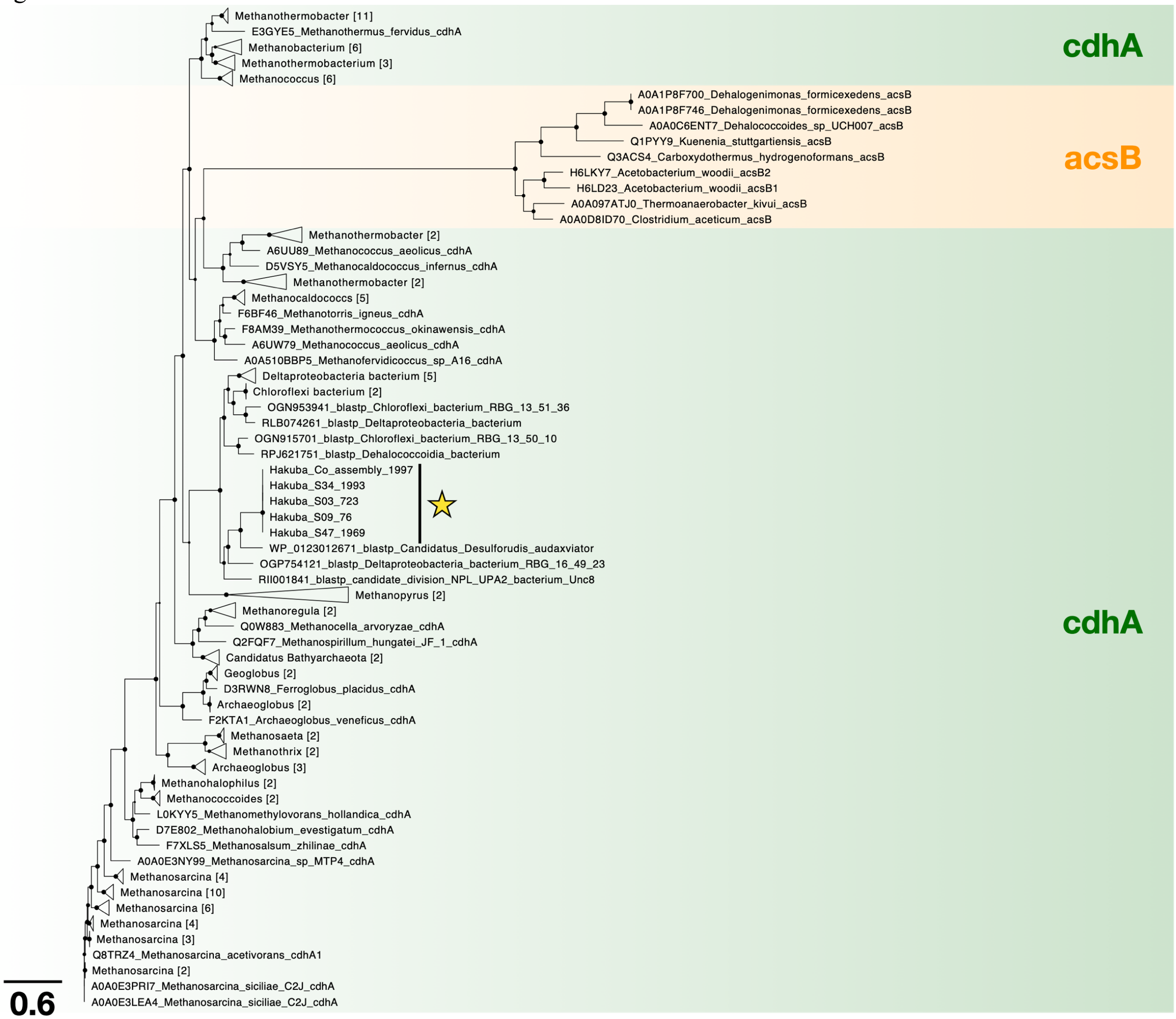

Figure S7B - CdhB

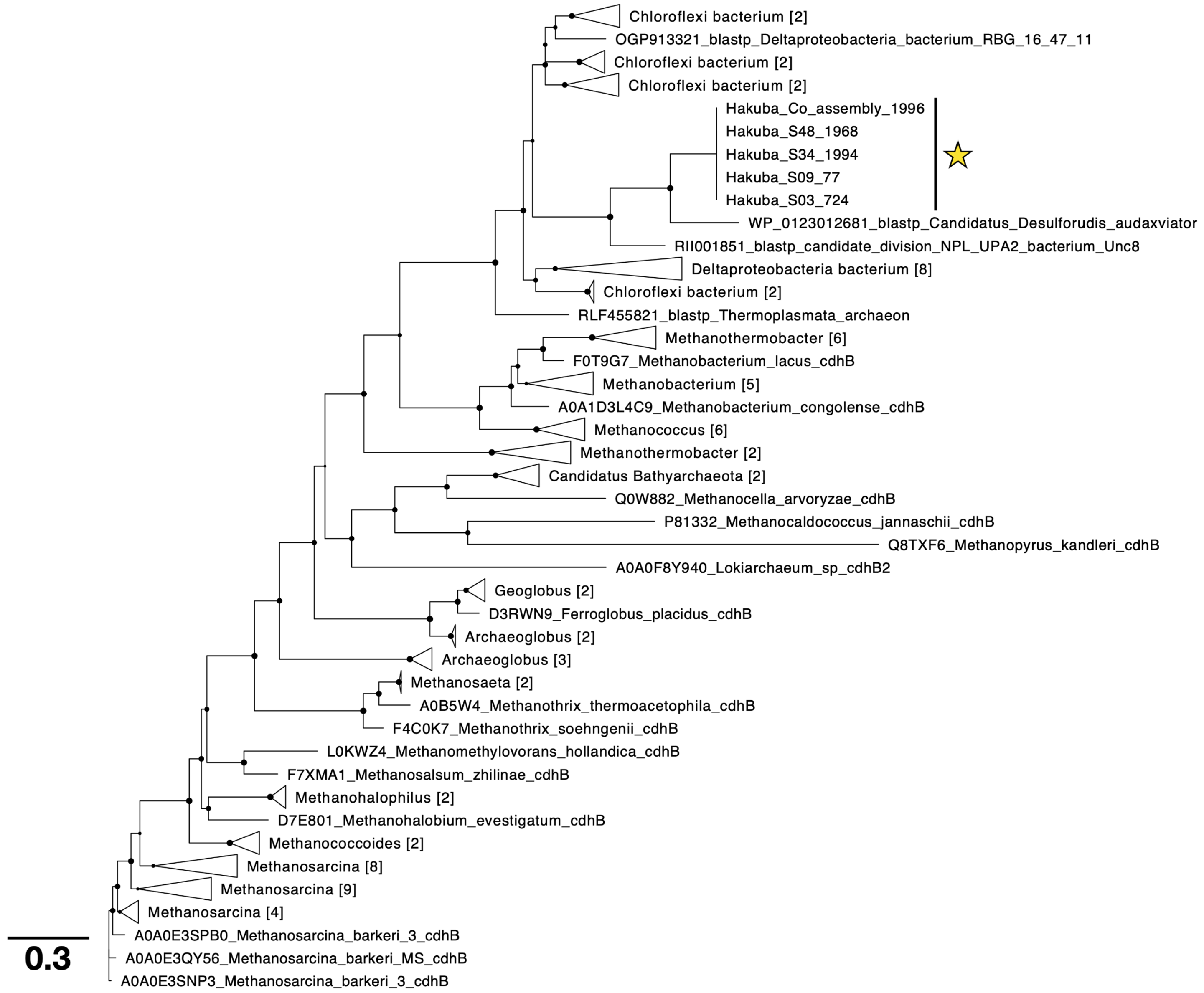

Figure S7C – CdhC/AcsA

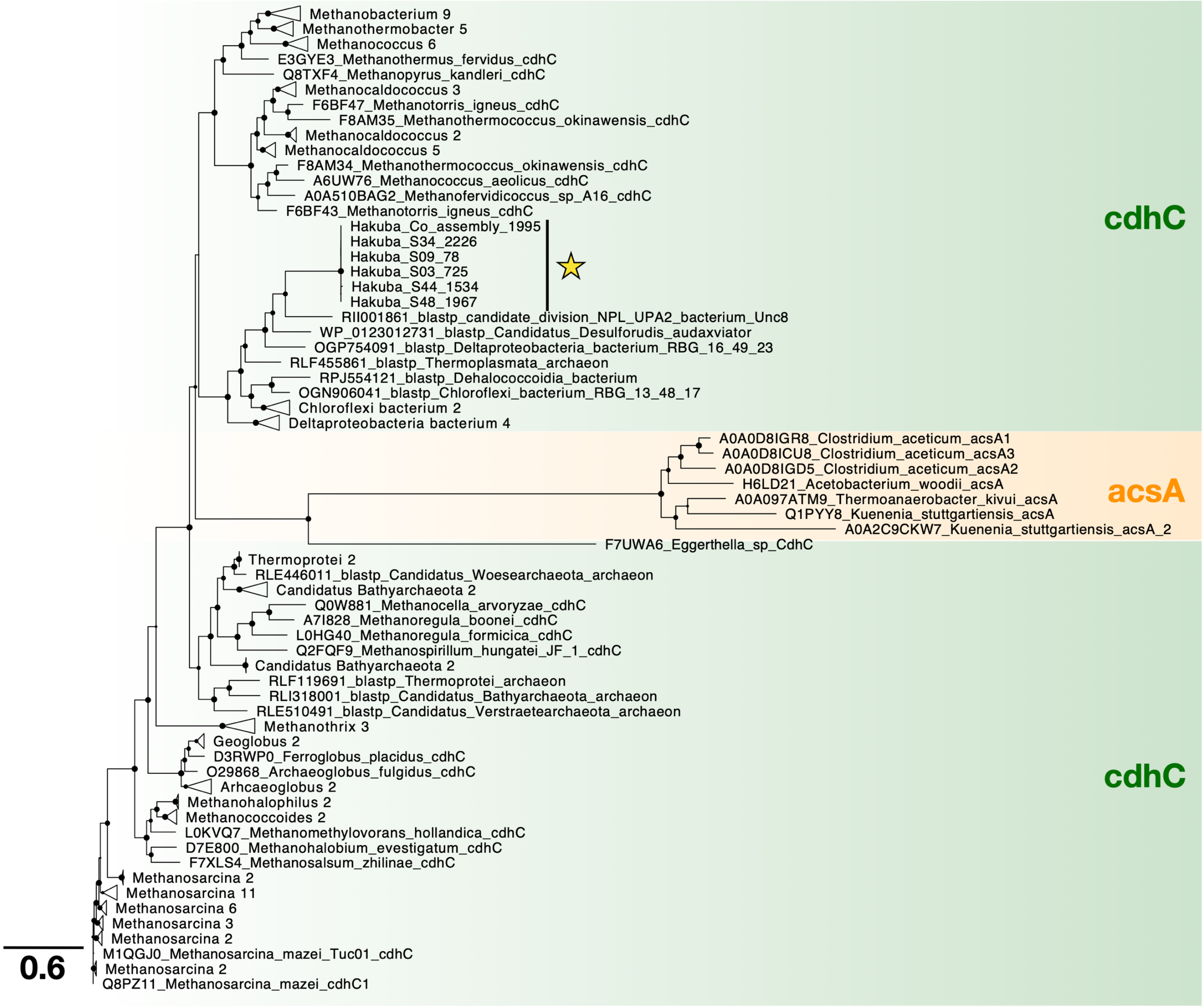

Figure S7D – AcsC/CdhE

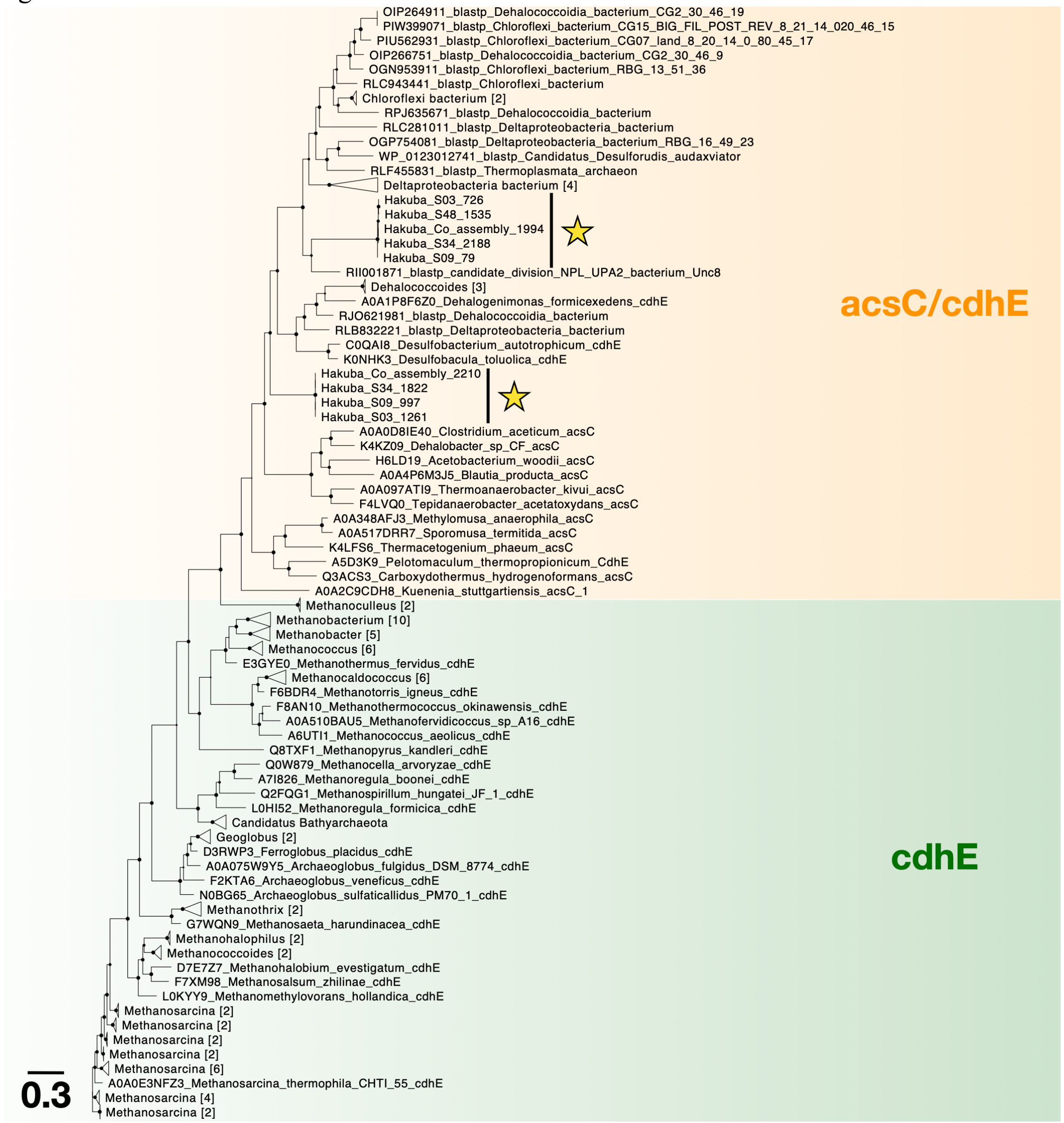

Figure S7E – AcsD/CdhD

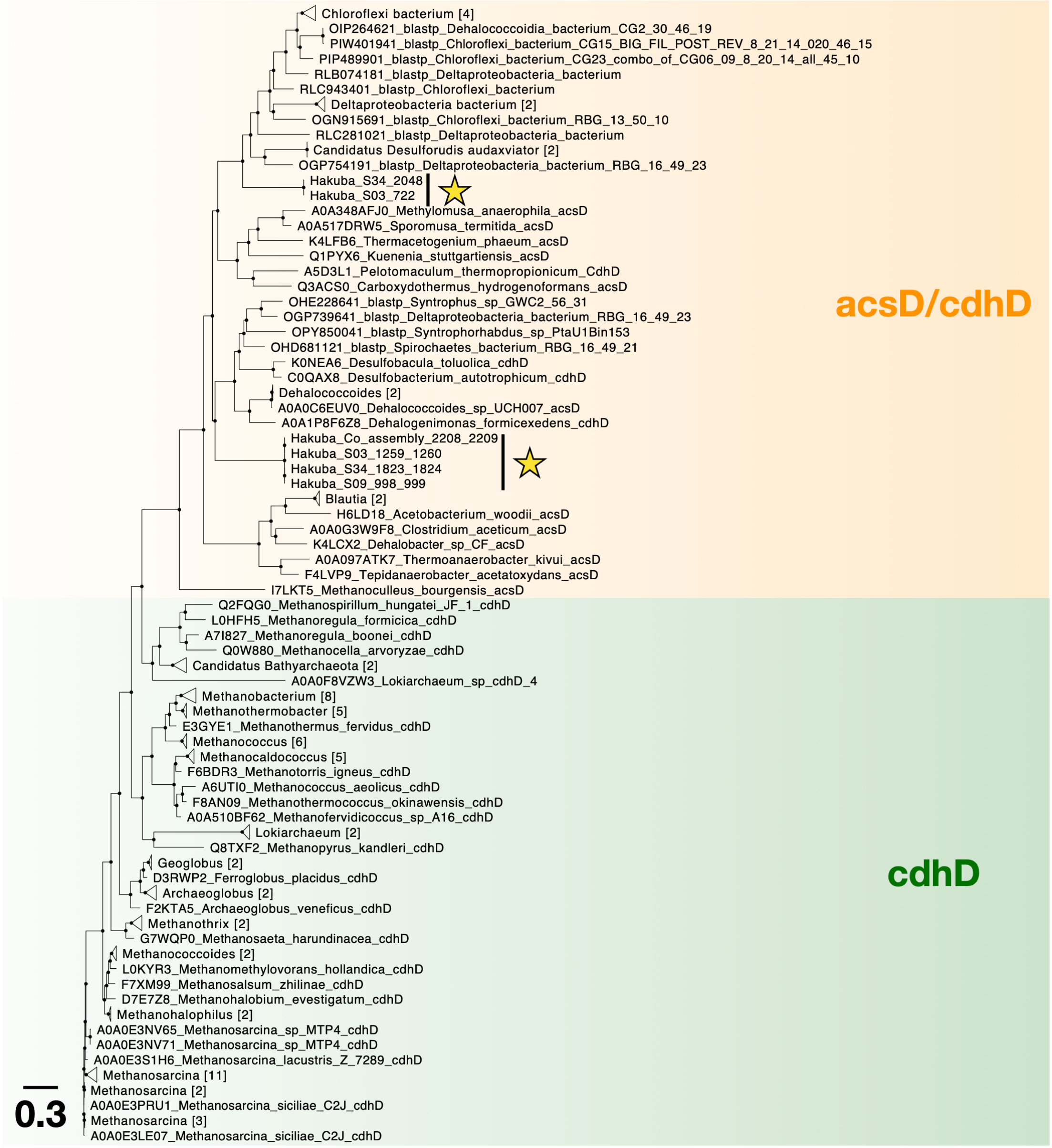

Figure S7F - AcsE

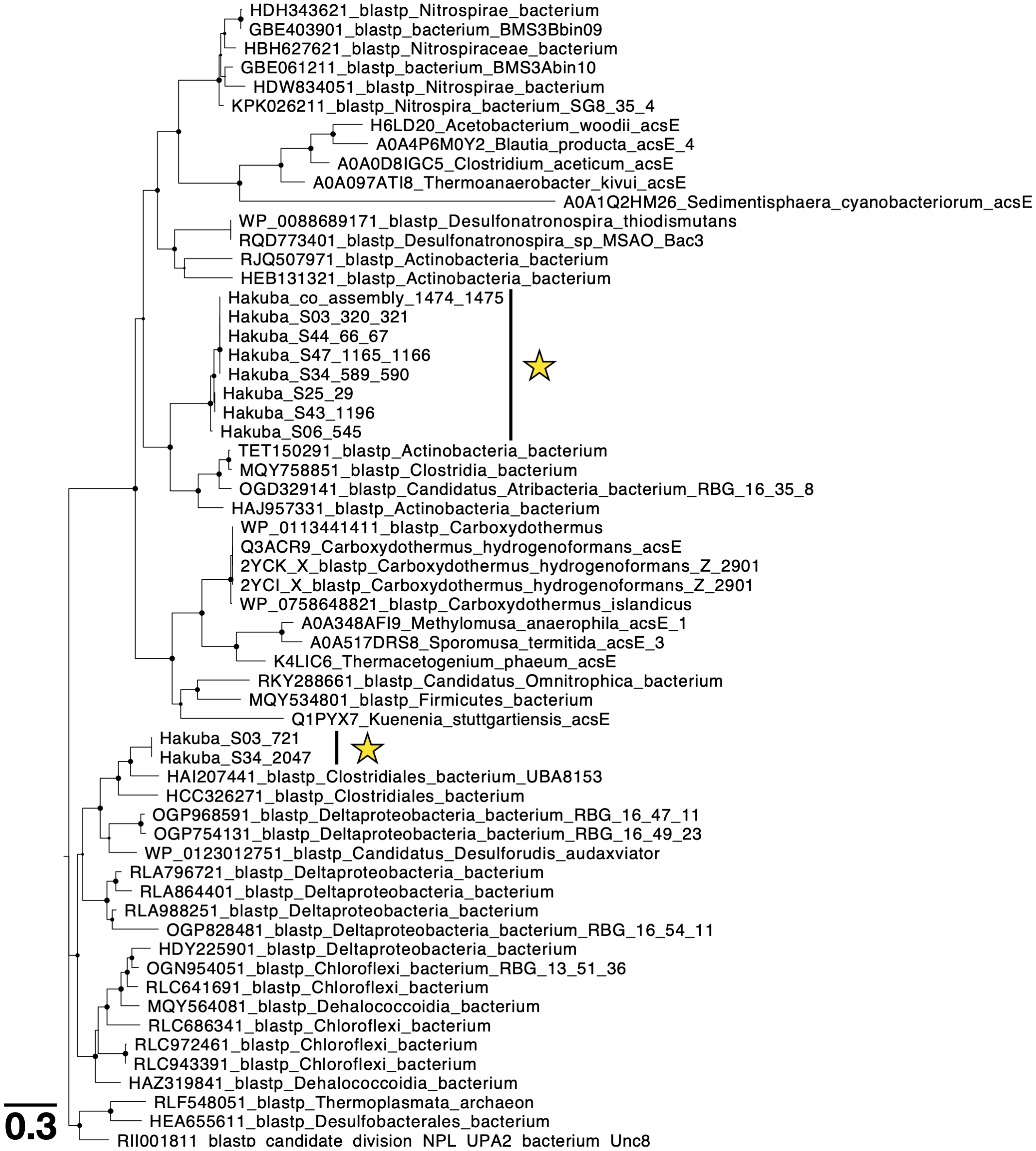

Figure S7. **Unrooted phylogenetic tree of CODH/ACS subunits.** Sequences for each subunit were obtained from UniProt database by searching the KEGG ID and gene name. Additional sequences (labeled with "blastp") were obtained using NCBI blastp against the appropriate subunit found in the Hakuba co-assembly and SAGs, and the top 20 hits were retrieved. Duplicate sequences (except for the Hakuba co-assembly and SAGs) were removed using Dedupe in BBTools (Bushnell et al., 2017). The Hakuba co-assembly and SAGs are denoted by a yellow star, and the number indicates gene ID (two gene IDs indicates a split gene that have been combined together to produce the phylogenetic tree). Archaeal subunits are colored green and bacterial subunits are colored orange. Black circles at nodes indicate support value, as calculated by FastTree (Price et al., 2010), and the size range from 0.2–1 for all subunits except for *cdhE*/*acsC* which ranges from 0–1. The number within "[]" indicates the number of genomes found in the collapsed clade, which was collapsed based on the same identification at the genus level.

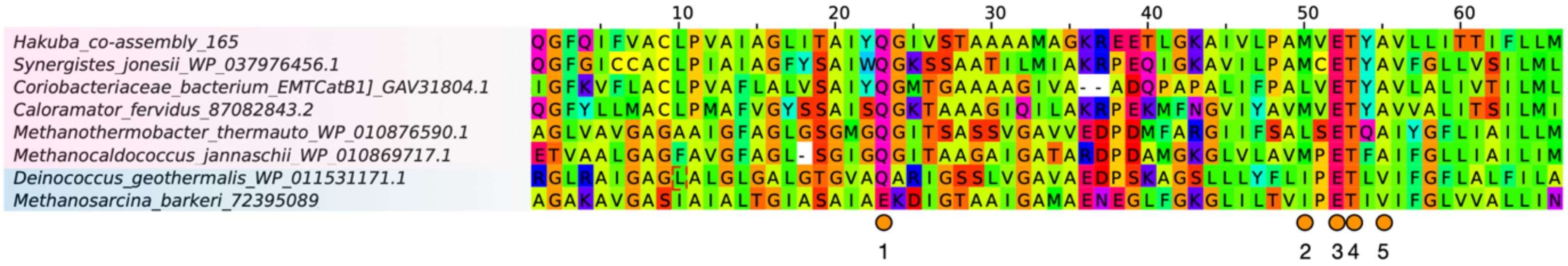

Figure S8. Multiple sequence alignment of the V-type ATPase subunit K transmembrane portion in the Hakuba co-assembly and select microorganisms. The transmembrane portion of V-type ATPase subunit K was aligned for microorganisms known to harbor Na^{+}-binding (pink color) and H^{+}-binding regions (blue color). The ion binding preference has been experimentally determined for *Caloramator fervidus*, *Methanothermobacter thermoauto*, *Methanocaldococcus jannaschii*, and *Methanosarcina barkeri*. The following microorganisms are closely related sequences to the Hakuba co-assembly V-type ATPase subunit K by NCBI BlastP: *Synergistes jonesii* and *Coriobacteriaceae* bacterium EMTCatB1]. The Na^{+}-binding conserved residues are identified with orange circles, where the conserved residues are (1) Q/E, (2) V/L/M/I, (3) E/Q, (4) S/T, and (5) A/G/S, according to Mulkidjanian et al. (2008).

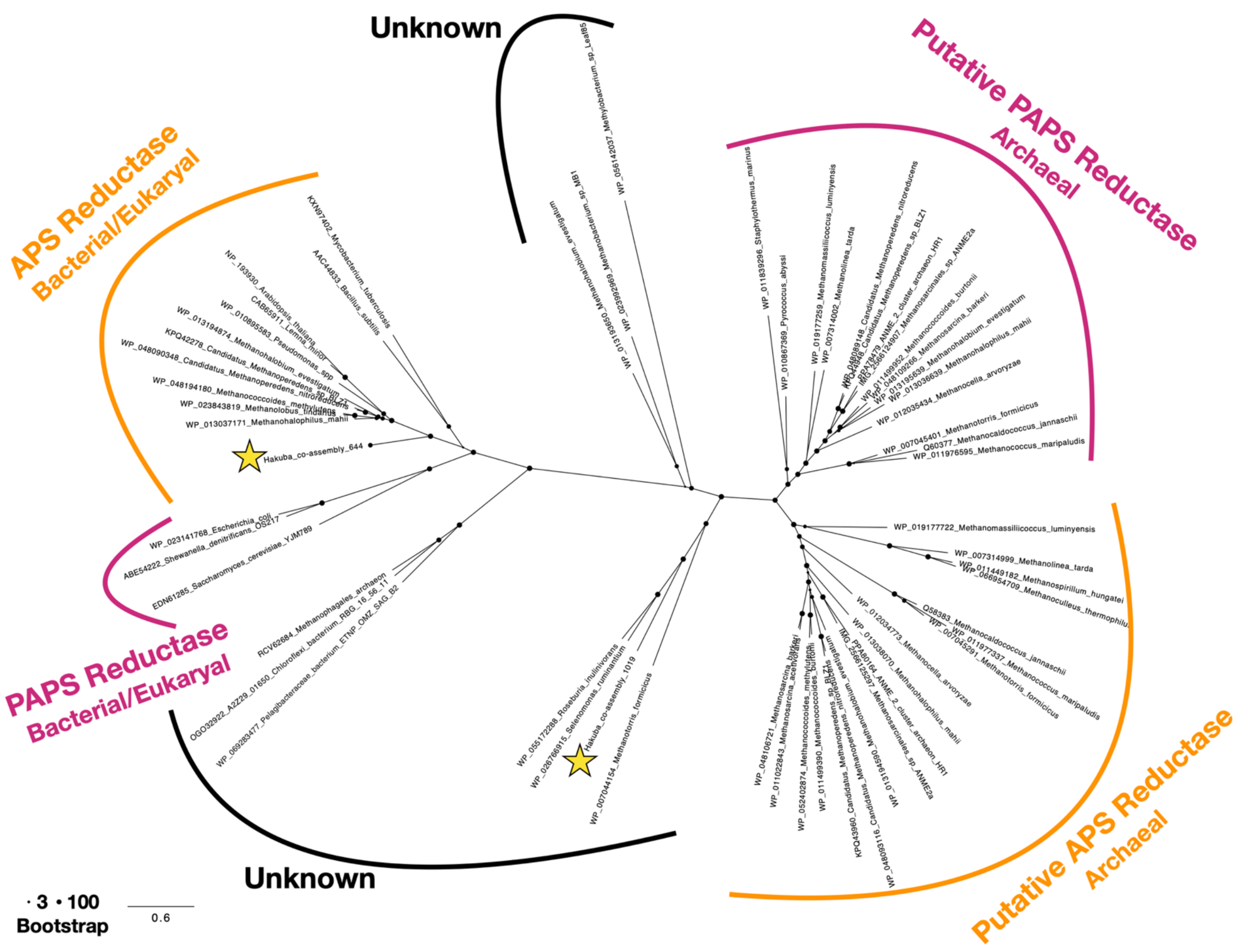

Figure S9. Unrooted phylogenetic tree of assimilatory adenyl-sulfate (APS) reductases and phosphoadenylyl-sulfate (PAPS) reductases. Sequences of APS (orange) and PAPS (purple) reductases were retrieved from Yu *et al.* (2018) and the Hakuba co-assembly (denoted by yellow star). Some genomes contain an APS or PAPS reductase with unknown substrate (labeled "Unknown").

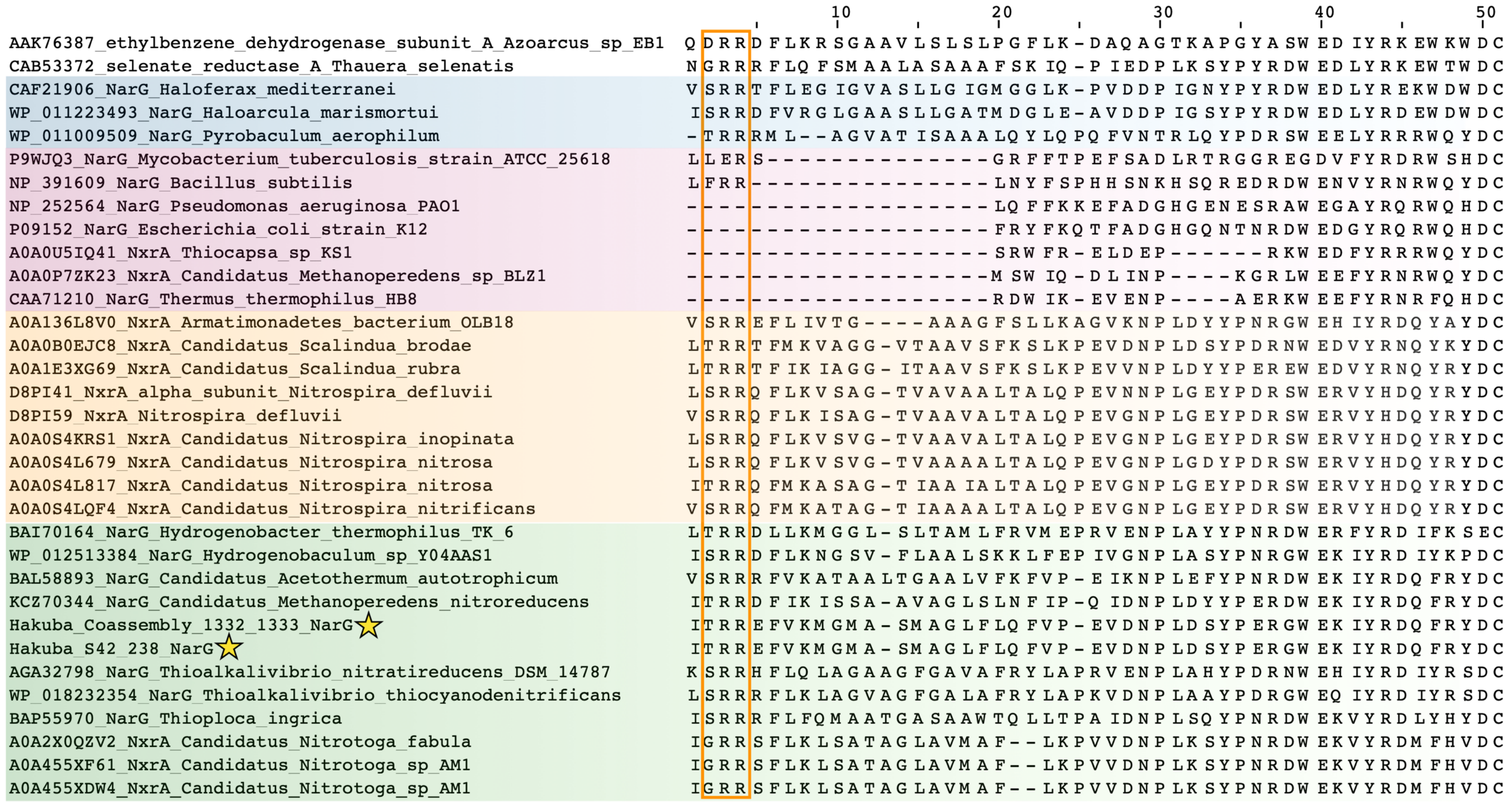

Figure S10. Alignment of N-terminal regions of NarG and NxrA and the conserved twin-arginine motif. The conserved twin-arginine motif [S/T]RR is shown in the orange box. The Hakuba co-assembly and SAG S42 is denoted by a yellow star and the numbers 1332, 1333, and 238 indicate the protein sequence ID. The color code for each clade follows Figure S11.

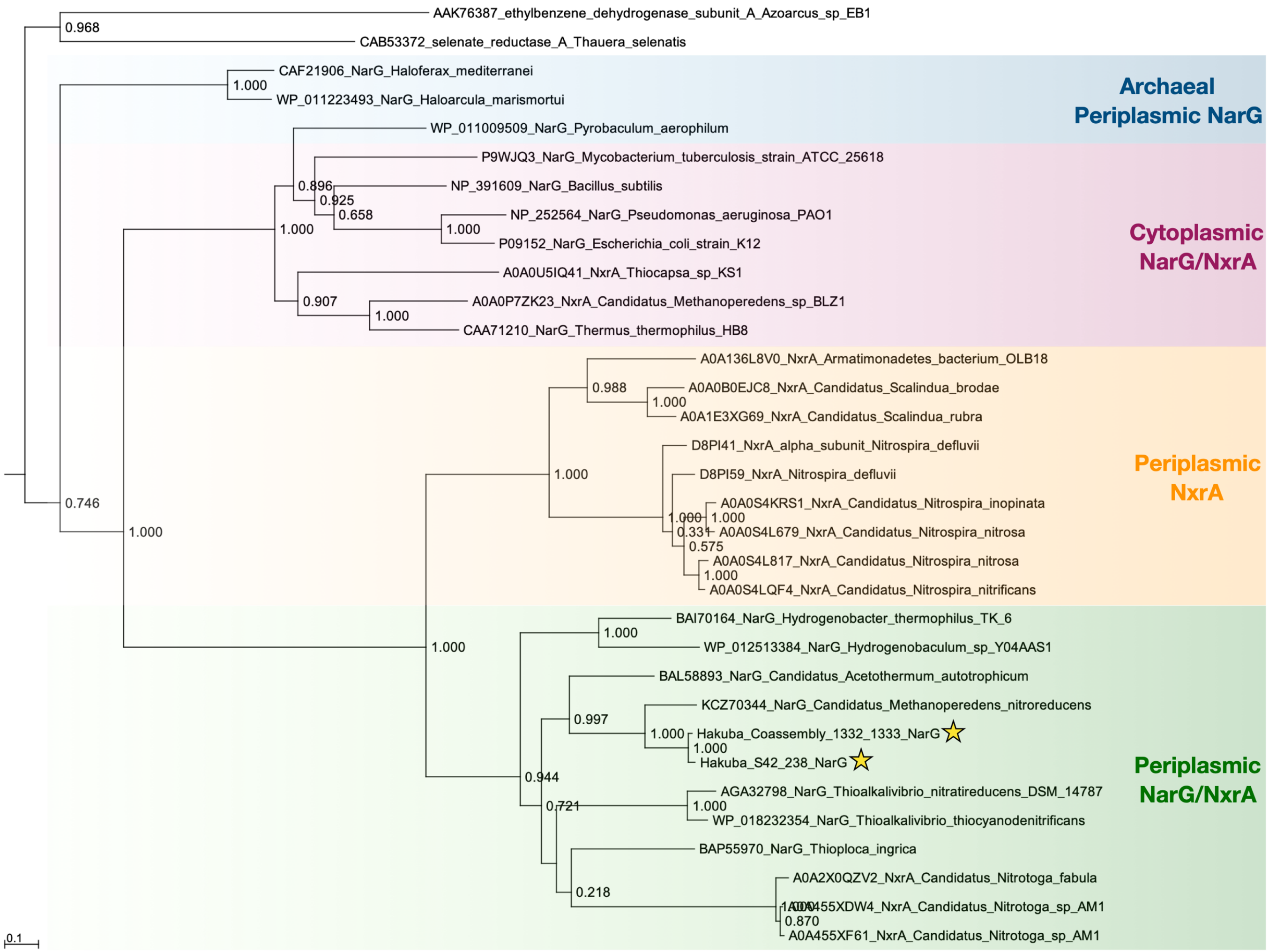

Figure S11. Maximum-likelihood phylogenetic tree of nitrate reductase alpha subunit NarG and NxrA. The phylogenetic tree was modified from Kameya *et al.* (2017) with additional sequences for nitrite oxidoreductase alpha subunit (NxrA) and reviewed sequences of NarG from the UniProt database. The Hakuba co-assembly and SAG S42 is denoted by a star. Sequences for ethylbenzene dehydrogenase and selenate reductase were used as the outgroups.

A. Amino acid sequence of *narG*

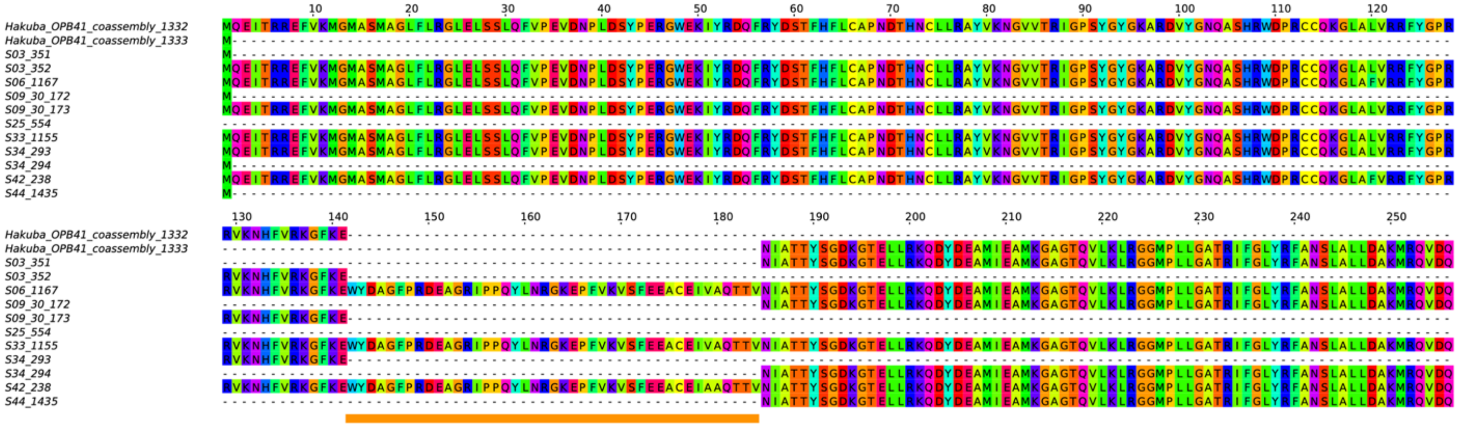

B. Nucleotide sequence of *narG*

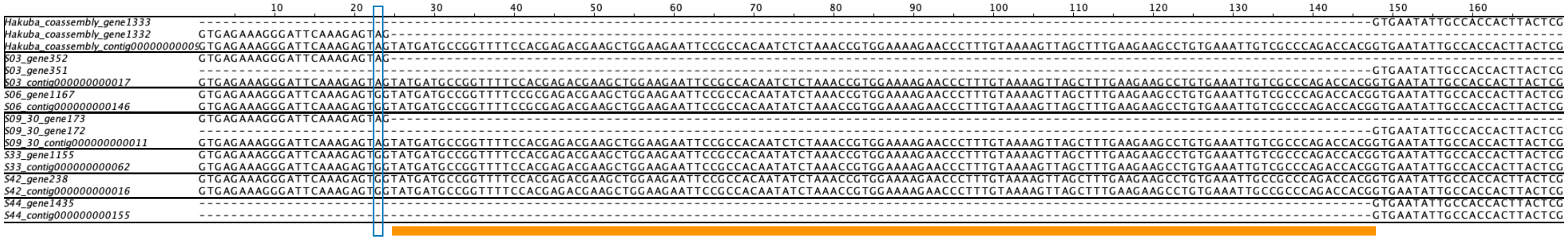

Figure S12. NarG alignment from the Hakuba SAGs and co-assembly. NarG amino acid and nucleotide sequences from the Hakuba SAGs and co-assembly were aligned. **(A)** The first 256 amino acid bases of the N-terminal region are depicted. In addition to the co-assembled genome, the SAGs S03, S09, and S34 have a split *narG* gene due to a nonsense mutation. The SAGs S06, S33, and S42 have the full-length *narG* sequence. The orange corresponds to the missing residues of the split *narG* gene from the Hakuba co-assembly and S03, S09, and S34. **(B)** The nucleotide alignment, highlighting the nonsense mutation (blue box). Depicted sequences correspond to 24 bp upstream and downstream of the sequence that corresponds to the missing residues of the amino-acid sequence (highlighted in orange).

Figure S13A

narG

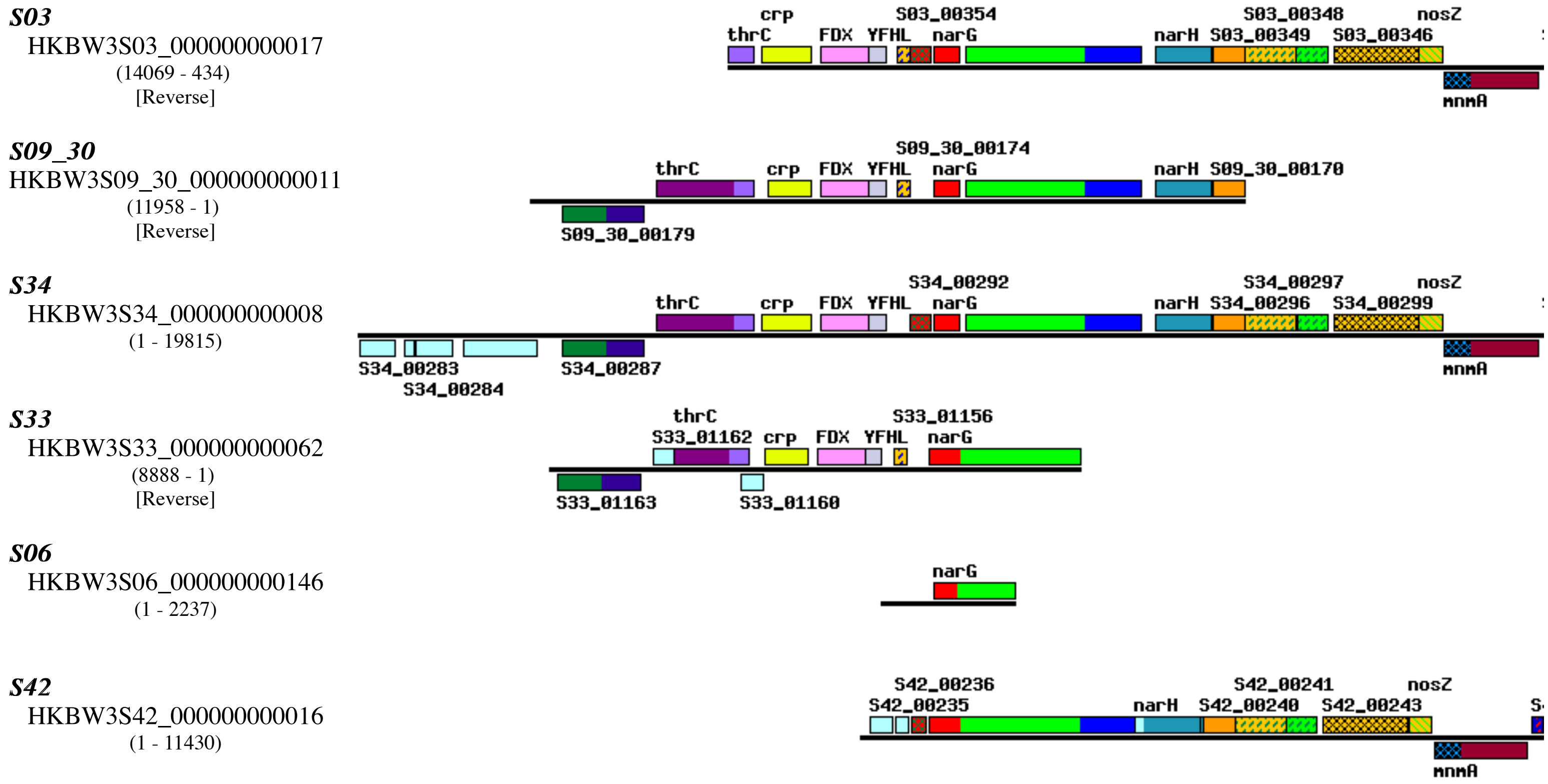

Figure S13B

nox1/hcaD

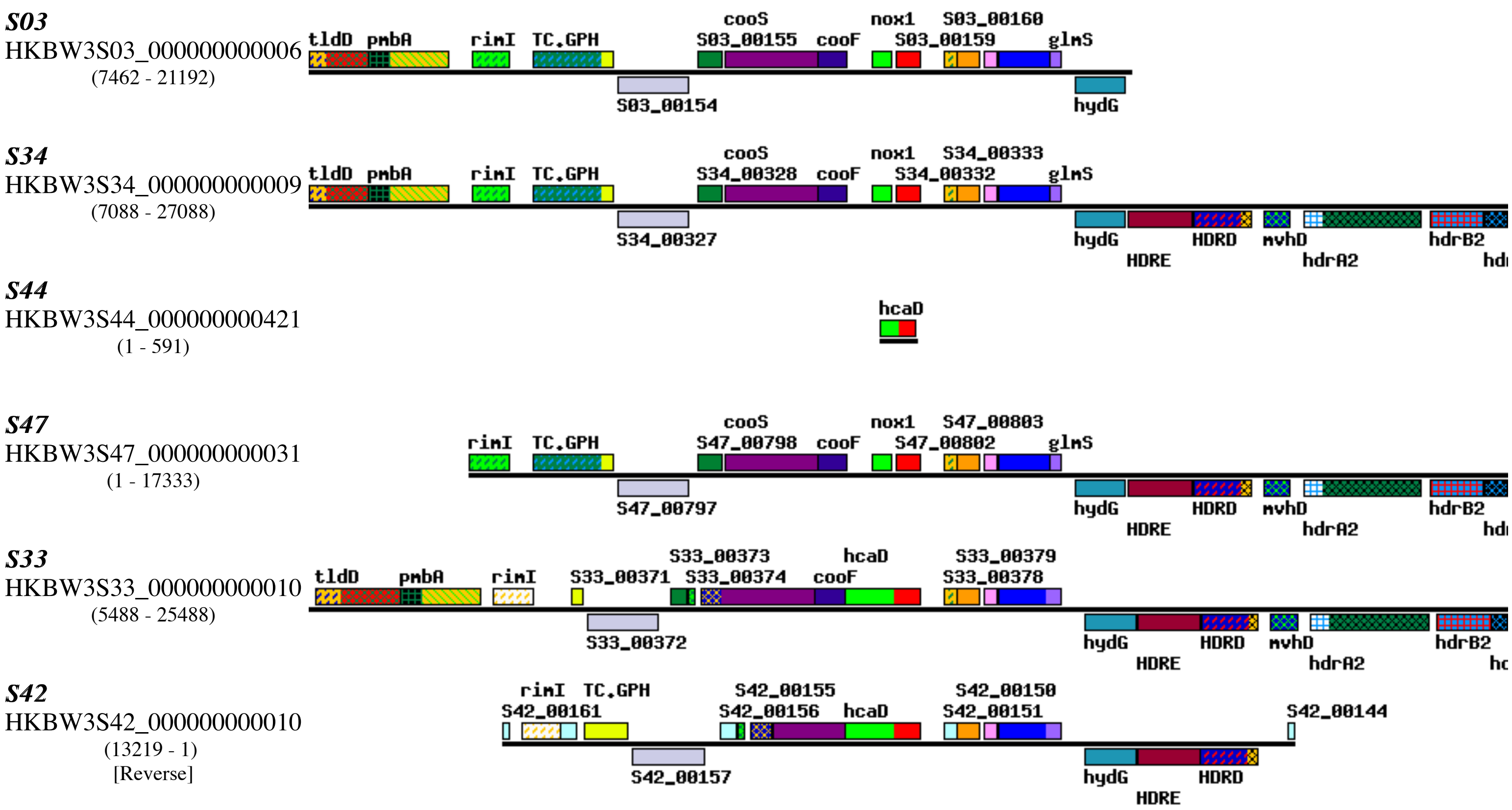

Figure S13C

acsE

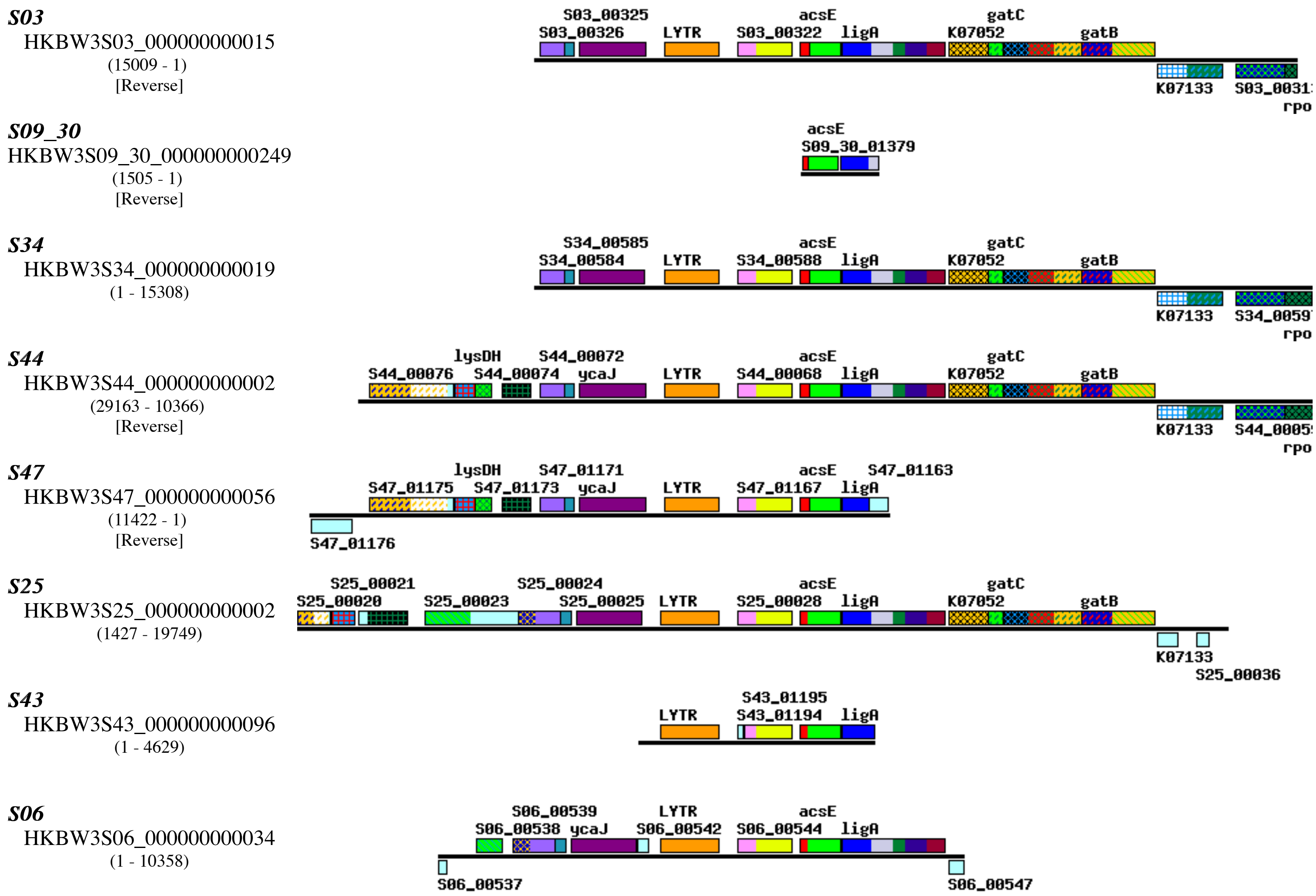

Figure S13D

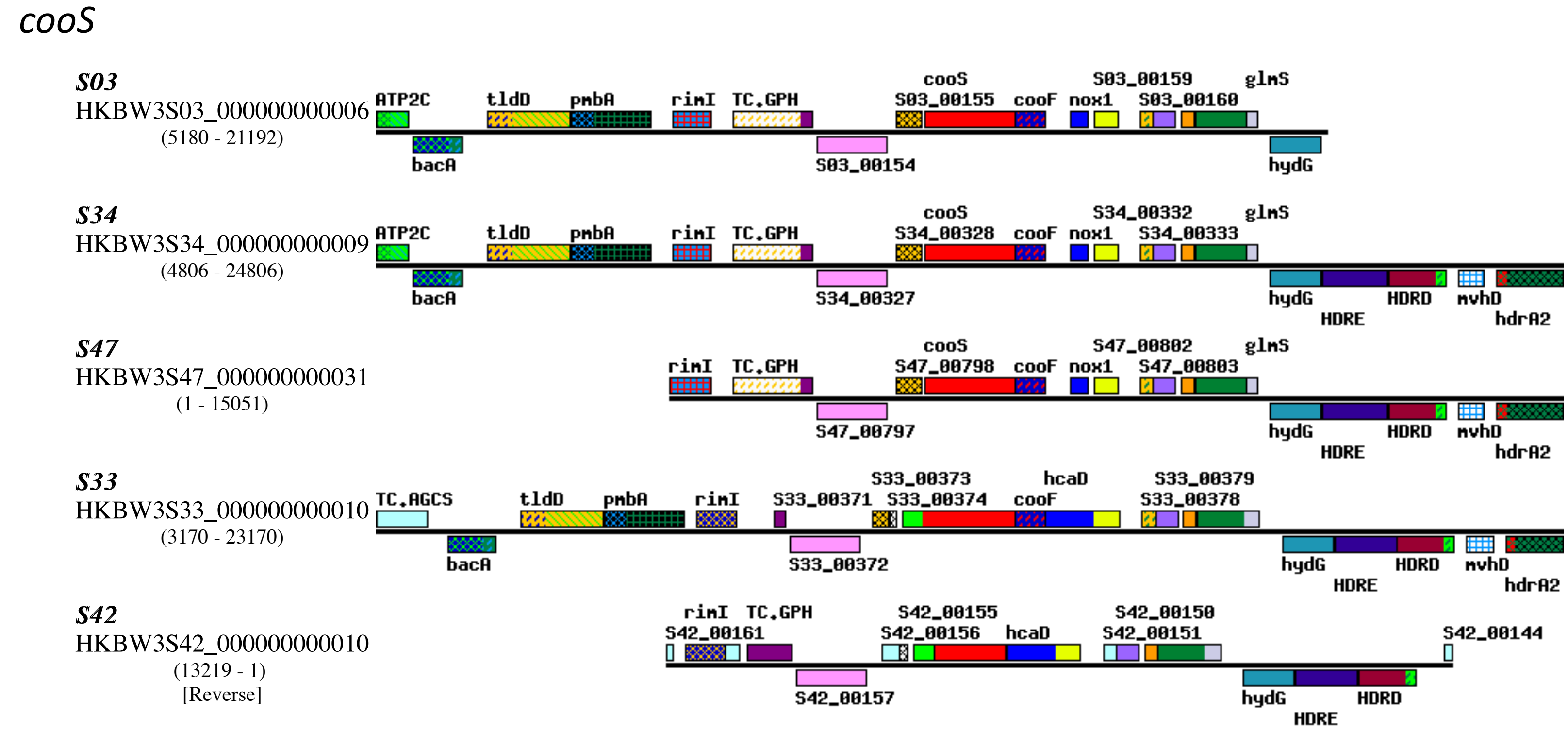
cooS
S03
HKBW3S03_000000000006
(5180 - 21192)
ATP2C
tldD
pmbA
rimI
TC.GPH
cooS
S03_00155
cooF
S03_00159
nox1
S03_00160
glmS
bacA
S03_00154
hydG
S34
HKBW3S34_000000000009
(4806 - 24806)
ATP2C
tldD
pmbA
rimI
TC.GPH
cooS
S34_00328
cooF
S34_00332
nox1
S34_00333
glmS
bacA
S34_00327
hydG
HDRE
HDRD
mvhD
hdrA2
S47
HKBW3S47_000000000031
(1 - 15051)
rimI
TC.GPH
cooS
S47_00798
cooF
S47_00802
nox1
S47_00803
glmS
S47_00797
hydG
HDRE
HDRD
mvhD
hdrA2
S33
HKBW3S33_000000000010
(3170 - 23170)
TC.AGCS
tldD
pmbA
rimI
S33_00371
S33_00373
S33_00374
hcaD
cooF
S33_00379
S33_00378
bacA
S33_00372
hydG
HDRE
HDRD
mvhD
hdrA2
S42
HKBW3S42_000000000010
(13219 - 1)
[Reverse]
rimI
S42_00161
TC.GPH
S42_00155
S42_00156
hcaD
S42_00150
S42_00151
S42_00144
S42_00157
hydG
HDRE
HDRD

Figure S13E

gas vesicle protein *gvpF*

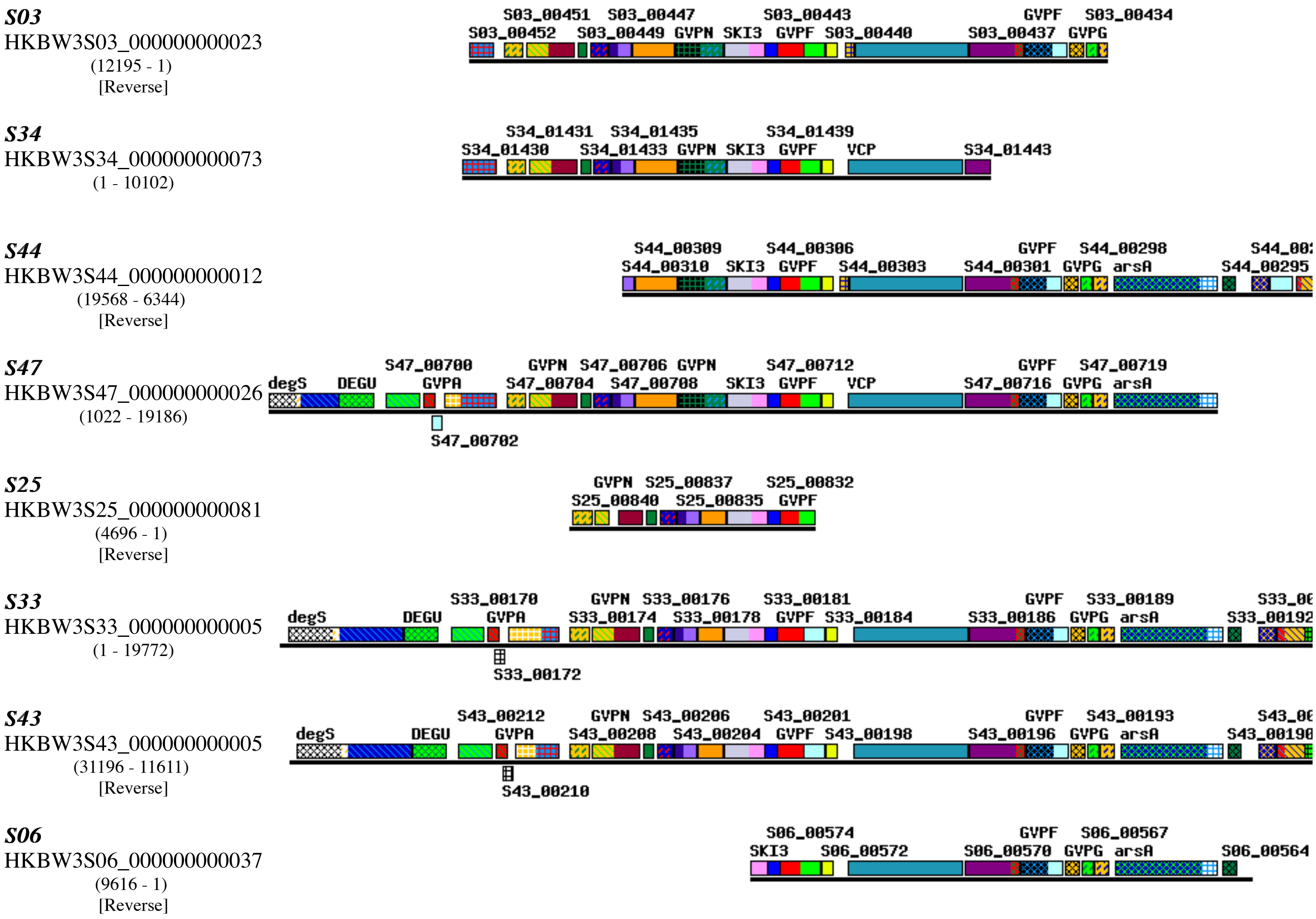

Figure S13. Multi-genome gene neighborhood of split genes and its corresponding members of the same orthologous group. Each figure depicts the gene neighborhood of every member (domains of genes) of an orthologous group (OG). The OG is depicted in red. Part of genes indicated in the same color or color-pattern except for light skyblue are members of one orthologous group. Flanking 10-kb regions at both sides of the member of the OG in red are depicted. OGs were generated by DomClust, a clustering program that identifies OGs, not only at the ORF level, but also at the domain level. This means one ORF can be split into multiple domains. (**A)** *narG* (S03, S09, S34 were a split type). (**B)** *nox1/hcaD* (S03, S34, and S47 were a split type and labeled as *nox1* whereas S33, S42, and S44 were a non-split type and labeled as *hcaD*). (**C)** *acsE* (S03, S09, S34, S44, and S47 were a split type). (**D)** *cooS* (S42 was a split type. Among the members of the non-split type, only S33 had another domain depicted in yellow green which corresponded to another gene of a split type in S42. The OG in yellow green was absent in S03, S34, and S47. (**E)** gas vesicle protein *gvpF* (S03, S33, and S43 were a split type). The OGs of each figure and genes that are members of the OGs are listed in **Table S12**. The information at the left side of each row of each figure is as follows: the SAG name, in bold and italicized, followed by the name of the contig of the genome, numbers in parentheses for the depicted range on the contig, the orientation depicted compared to the deposited orientation of the sequence of the contig, when reverse strand is depicted.

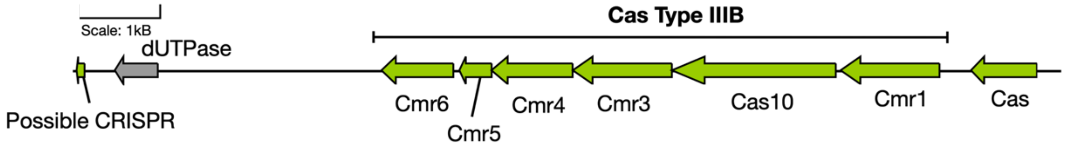

Figure S14. CRISPR/Cas gene cluster in the Hakuba co-assembly. CRISPR/Cas genes (green) were identified in contig 000000000051, as determined by CRISPRCasFinder (Couvin et al., 2018). The *cas* genes are composed of a *cas* type-IIIB gene cluster and one putative *cas* gene. A possible CRISPR sequence (CRISPRCasFinder evidence level = 1) was also detected and is a 99 nucleotide bp segment with two repeat sequences (CTTAGGTATCGGTCTCCTTTCTCA) and one spacer (TGGTTTGACCCATATAGCTATTAGGCATGGTTAGCCTCCGTTTCAATCCCT) that does not resemble any viral sequences in the CRISPRCasFinder database or the NCBI nr database. Gene neighborhood was designed using Gene Graphics (Harrison et al., 2017).

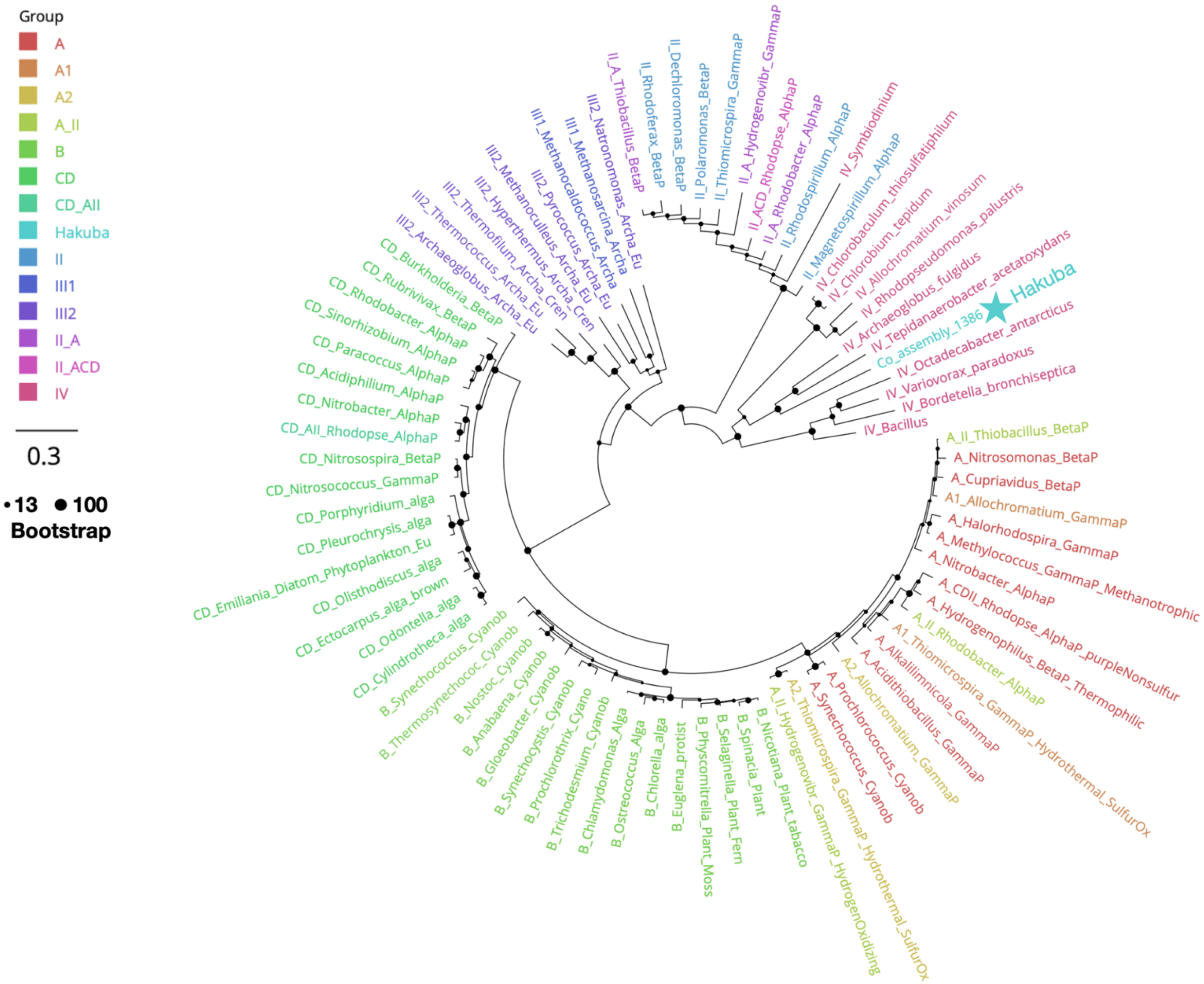

Figure S15. Maximum-likelihood phylogenetic tree of all RubisCO forms. A ML phylogenetic tree was created by utilizing sequences from Kacar et al. (2017) and including the Hakuba co-assembly RubisCO-like protein sequence (denoted with turquoise star). RAxML (Gamma model) on the CIPRES Science Gateway was used to obtain the bootstrap values.

References

Adam, P. S., Borrel, G., and Gribaldo, S. (2019). An archaeal origin of the Wood–Ljungdahl H_4MPT branch and the emergence of bacterial methylotrophy. *Nat Microbiol* 4, 2155–2163. doi:10.1038/s41564-019-0534-2.

Bateman, A., Martin, M. J., O'Donovan, C., Magrane, M., Alpi, E., Antunes, R., et al. (2017). UniProt: the universal protein knowledgebase. *Nucleic Acids Res* 45, D158–D169. doi:10.1093/nar/gkw1099.

Bujdák, J., and Rode, B. M. (1999). The effect of clay structure on peptide bond formation catalysis. *J Mol Catal A-Chem* 144, 129–136. doi:10.1016/S1381-1169(98)00342-2.

Bushnell, B., Rood, J., and Singer, E. (2017). BBMerge – Accurate paired shotgun read merging via overlap. *PLoS ONE* 12, e0185056. doi:10.1371/journal.pone.0185056.

Callahan, B. J., McMurdie, P. J., Rosen, M. J., Han, A. W., Johnson, A. J. A., and Holmes, S. P. (2016). DADA2: High-resolution sample inference from Illumina amplicon data. *Nat Methods* 13, 581–583. doi:10.1038/nmeth.3869.

Camacho, C., Coulouris, G., Avagyan, V., Ma, N., Papadopoulos, J., Bealer, K., et al. (2009). BLAST+: architecture and applications. *BMC Bioinformatics* 10, 421. doi:10.1186/1471-2105-10-421.

Capella-Gutierrez, S., Silla-Martinez, J. M., and Gabaldon, T. (2009). trimAl: a tool for automated alignment trimming in large-scale phylogenetic analyses. *Bioinformatics* 25, 1972–1973. doi:10.1093/bioinformatics/btp348.

Carroll, K. S., Gao, H., Chen, H., Stout, C. D., Leary, J. A., and Bertozzi, C. R. (2005). A Conserved Mechanism for Sulfonucleotide Reduction. *PLoS Biol* 3, e250. doi:10.1371/journal.pbio.0030250.

Couvin, D., Bernheim, A., Toffano-Nioche, C., Touchon, M., Michalik, J., Néron, B., et al. (2018). CRISPRCasFinder, an update of CRISRFinder, includes a portable version, enhanced performance and integrates search for Cas proteins. *Nucleic Acids Res* 46, W246–W251. doi:10.1093/nar/gky425.

Eren, A. M., Esen, Ö. C., Quince, C., Vineis, J. H., Morrison, H. G., Sogin, M. L., et al. (2015). Anvi'o: an advanced analysis and visualization platform for 'omics data. *PeerJ* 3, e1319. doi:10.7717/peerj.1319.

Garber, A., Nealson, K. H., Okamoto, A., Chan, C. S., McAllister, S. M., and Merino, N. (2019). FeGenie: a comprehensive tool for the identification of iron genes and iron gene clusters in genomes and metagenome assemblies. *bioRxiv*. doi:doi.org/10.1101/777656.

Goudeau, D., Nath, N., Ciobanu, D., Cheng, J.-F., and Malmstrom, R. (2014). Current Developments in Prokaryotic Single Cell Whole Genome Amplification.

Harrison, K. J., de Crecy-Lagard, V., and Zallot, R. (2017). Gene Graphics: a genomic neighborhood data visualization web application. *Bioinformatics* 34, 1406–1408. doi:10.1093/bioinformatics/btx793.

Hemmann, J. L., Wagner, T., Shima, S., and Vorholt, J. A. (2019). Methylofuran is a prosthetic group of the formyltransferase/hydrolase complex and shuttles one-carbon units between two active sites. *Proc Natl Acad Sci USA*, 201911595. doi:10.1073/pnas.1911595116.

Kacar, B., Hanson-Smith, V., Adam, Z. R., and Boekelheide, N. (2017). Constraining the timing of the Great Oxidation Event within the Rubisco phylogenetic tree. *Geobiology* 15, 628–640. doi:10.1111/gbi.12243.

Kameya, M., Kanbe, H., Igarashi, Y., Arai, H., and Ishii, M. (2017). Nitrate reductases in *Hydrogenobacter thermophilus* with evolutionarily ancient features: distinctive localization and electron transfer: Nitrate reductases of *Hydrogenobacter thermophilus*. *Mol Microbiol* 106, 129–141. doi:10.1111/mmi.13756.

Katoh, K., Misawa, K., Kuma, K., and Miyata, T. (2002). MAFFT: a novel method for rapid multiple sequence alignment based on fast Fourier transform. *Nucleic Acids Res* 30, 3059–3066. doi:10.1093/nar/gkf436.

Kawai, M., Furuta, Y., Yahara, K., Tsuru, T., Oshima, K., Handa, N., et al. (2011). Evolution in an oncogenic bacterial species with extreme genome plasticity: Helicobacter pylori East Asian genomes. *BMC Microbiol* 11, 104. doi:10.1186/1471-2180-11-104.

Kawai, M., Futagami, T., Toyoda, A., Takaki, Y., Nishi, S., Hori, S., et al. (2014). High frequency of phylogenetically diverse reductive dehalogenase-homologous genes in deep subseafloor sedimentary metagenomes. *Front Microbiol* 5. doi:10.3389/fmicb.2014.00080.

Lasken, R. S., and Stockwell, T. B. (2007). Mechanism of chimera formation during the Multiple Displacement Amplification reaction. *BMC Biotechnol* 7, 19. doi:10.1186/1472-6750-7-19.

Lux, M., Krüger, J., Rinke, C., Maus, I., Schlüter, A., Woyke, T., et al. (2016). acdc – Automated Contamination Detection and Confidence estimation for single-cell genome data. *BMC Bioinformatics* 17, 543. doi:10.1186/s12859-016-1397-7.

Menzel, P., Ng, K. L., and Krogh, A. (2016). Fast and sensitive taxonomic classification for metagenomics with Kaiju. *Nat Commun* 7, 11257. doi:10.1038/ncomms11257.

Mistry, J., Finn, R. D., Eddy, S. R., Bateman, A., and Punta, M. (2013). Challenges in homology search: HMMER3 and convergent evolution of coiled-coil regions. *Nucleic Acids Res* 41, e121–e121. doi:10.1093/nar/gkt263.

Mulkidjanian, A. Y., Galperin, M. Y., Makarova, K. S., Wolf, Y. I., and Koonin, E. V. (2008). Evolutionary primacy of sodium bioenergetics. *Biol Direct* 3, 13. doi:10.1186/1745-6150-3-13.

Ohara, S., Kakegawa, T., and Nakazawa, H. (2007). Pressure Effects on the Abiotic Polymerization of Glycine. *Orig Life Evol Biosph* 37, 215–223. doi:10.1007/s11084-007-9067-4.

Pomper, B. K., Saurel, O., Milon, A., and Vorholt, J. A. (2002). Generation of formate by the formyltransferase/hydrolase complex (Fhc) from *Methylobacterium extorquens* AM1. *FEBS Letters* 523, 133–137. doi:10.1016/S0014-5793(02)02962-9.

Price, M. N., Dehal, P. S., and Arkin, A. P. (2010). FastTree 2 – Approximately Maximum-Likelihood Trees for Large Alignments. *PLoS ONE* 5, e9490. doi:10.1371/journal.pone.0009490.

Rost, B. (1999). Twilight zone of protein sequence alignments. *Protein Eng Des Sel* 12, 85–94. doi:10.1093/protein/12.2.85.

Steinegger, M., and Söding, J. (2017). MMseqs2 enables sensitive protein sequence searching for the analysis of massive data sets. *Nature Biotechnology*. doi:10.1038/nbt.3988.

Uchiyama, I. (2006). Hierarchical clustering algorithm for comprehensive orthologous-domain classification in multiple genomes. *Nucleic Acids Res* 34, 647–658. doi:10.1093/nar/gkj448.

Uchiyama, I. (2017). Ortholog Identification and Comparative Analysis of Microbial Genomes Using MBGD and RECOG. *Methods Mol. Biol.* 1611, 147–168. doi:10.1007/978-1-4939-7015-5_12.

Yu, H., Susanti, D., McGlynn, S. E., Skennerton, C. T., Chourey, K., Iyer, R., et al. (2018). Comparative Genomics and Proteomic Analysis of Assimilatory Sulfate Reduction Pathways in Anaerobic Methanotrophic Archaea. *Front Microbiol* 9, 2917. doi:10.3389/fmicb.2018.02917.
